# A Hierarchical Mechanism of RIG-I Ubiquitination Provides Sensitivity, Robustness and Synergy in Antiviral Immune Responses

**DOI:** 10.1038/srep29263

**Published:** 2016-07-08

**Authors:** Xiaoqiang Sun, Huifang Xian, Shuo Tian, Tingzhe Sun, Yunfei Qin, Shoutao Zhang, Jun Cui

**Affiliations:** 1 Zhong-shan School of Medicine, Sun Yat-sen University, Guangzhou 510089, China; 2 School of Life Science, Sun Yat-sen University, Guangzhou, 510275, China; 3 School of Mathematical and Computational Science, Sun Yat-sen University, Guangzhou, 510000, China; 4 School of Life Sciences, AnQing Normal University, AnQing, 246011, China; 5 School of Life Sciences, Zhengzhou University, Zhengzhou, Henan, 450001, China; 6 Collaborative Innovation Center of Cancer Medicine, Sun Yat-sen University, Guangzhou, 510060, China

## Abstract

RIG-I is an essential receptor in the initiation of the type I interferon (IFN) signaling pathway upon viral infection. Although K63-linked ubiquitination plays an important role in RIG-I activation, the optimal modulation of conjugated and unanchored ubiquitination of RIG-I as well as its functional implications remains unclear. In this study, we determined that, in contrast to the RIG-I CARD domain, full-length RIG-I must undergo K63-linked ubiquitination at multiple sites to reach full activity. A systems biology approach was designed based on experiments using full-length RIG-I. Model selection for 7 candidate mechanisms of RIG-I ubiquitination inferred a hierarchical architecture of the RIG-I ubiquitination mode, which was then experimentally validated. Compared with other mechanisms, the selected hierarchical mechanism exhibited superior sensitivity and robustness in RIG-I-induced type I IFN activation. Furthermore, our model analysis and experimental data revealed that TRIM4 and TRIM25 exhibited dose-dependent synergism. These results demonstrated that the hierarchical mechanism of multi-site/type ubiquitination of RIG-I provides an efficient, robust and optimal synergistic regulatory module in antiviral immune responses.

Innate immune responses are the first line of defense against invading pathogens^1^. Effective immune resistance to pathogenic micro-organisms depends on the efficiency of pathogen recognition by innate immune receptors. Retinoic-acid-inducible gene-I (RIG-I), one of these receptors, is an important viral RNA sensor that specifically recognizes short double-strand viral RNA (dsRNA) and activates the type I interferon (IFN) signaling pathway^2^.

RIG-I, melanoma differentiation factor 5 (MDA5) and laboratory of genetics and physiology 2 (LGP2) belong to the DExD/H-box family of helicases^2^. The RIG-I protein can be divided into three distinct domains: a domain containing two caspase recruitment domains (2CARDs), a helicase domain and a repressor domain (RD). 2CARD (aa 1–200), the N-terminal domain of RIG-I, is the functional domain that activates the type I IFN signaling pathway without dsRNA stimulation. RD (aa 735–925) can bind 2CARD to inhibit its function. The C-terminal domain (CTD) (aa 792–925) overlaps with the RD of RIG-I and recognizes the viral dsRNA and leads to the conformational change of RIG-I^3^. RIG-I activation is a complex process. RIG-I is distributed in the cytoplasm in an inactive form and undergoes the conformational rearrangement to expose the functional 2CARD domain only after recognizing the 5′-triphosphate dsRNA by CTD^4^. The subsequent dephosphorylation of RIG-I promotes the ubiquitination of RIG-I-2CARD^5^. Ubiquitinated RIG-I assembles into tetramers and then interacts with downstream adaptor mitochondrial antiviral signaling (MAVS), which transmits the activation signaling to activate the type I IFN signaling pathway^2^.

Ubiquitination plays crucial roles in RIG-I activation as well as the type I IFN signaling pathway^6^. Since Gack *et al.* reported that K63-linked ubiquitination of RIG-I-2CARD plays an indispensable role in RIG-I function^7^, crystal structure and functional analyses have revealed that conjugation of K63-linked ubiquitin chains to lysines are compatible with its unanchored binding to RIG-I-2CARD, and may further stabilize the RIG-I-2CARD tetramers^8^. Subsequent studies have focused on the role of unanchored ubiquitin chains, which are generated by E2/E3 ligases and are not covalently conjugated to a substrate protein^9^,^10^. In brief, K63-linked ubiquitination contributes substantially to RIG-I activation and downstream signaling.

TRIM25, TRIM4 and MEX3C are the most important E3 ligases for RIG-I-2CARD K63-linked ubiquitination^7^,^11^,^12^. Although the ubiquitination of RD by Riplet is obligatory^13^, most studies have focused on the ubiquitination of RIG-I-2CARD. RIG-I-2CARD can be ubiquitinated at lysines (K) 48, 99, 154, 164, 169, 172, 181, 190 and 193. Among these sites, K164 and K172 are most important ubiquitination sites for RIG-I-2CARD to activate type I IFN signaling^7^,^11^,^12^.

Although many regulators of RIG-I ubiquitination have been identified, how this multi-site/type ubiquitination optimally and cooperatively modulates RIG-I activation remains unclear. The ordered mechanism in multisite protein modification^14^ is an important means of regulating protein functions, such as ultrasensitivity and bistability^15^,^16^,^17^. Therefore, in this study, we sought to determine the mechanism by which the regulation of RIG-I ubiquitination by anchored and unanchored ubiquitin chains is involved in antiviral immune response. Furthermore, we examined the functional implications of multi-site/type ubiquitination of RIG-I in the innate immune response.

A rational and systematic analysis is required to analyze this complex signaling system. We designed a systems biology approach to quantitatively investigate multi-site/type ubiquitination of RIG-I and to explore the mechanisms of reaction order underlying RIG-I ubiquitination during the innate immune response. Unexpectedly, we determined that only double mutation and not single mutations of both the K164 and K172 sites of full-length RIG-I (FL-RIG-I) significantly blocked the activation of type I IFN signaling. We then proposed seven candidate mechanisms for FL-RIG-I ubiquitination and constructed a mathematical model for each mechanism. We fitted each model to the experimental data and inferred a hierarchical mechanism of FL-RIG-I ubiquitination using a model selection method. Importantly, we then experimentally validated the selected hierarchical mechanism. To understand the physiological significance of FL-RIG-I multi-site/type ubiquitination, we further quantitatively analyzed the dose-response sensitivity and robustness of each model. The results demonstrated that the hierarchical mechanism was superior as a compromise between sensitivity and robustness during the immune response. Furthermore, our model analysis suggested that TRIM4 and TRIM25 exhibited dose-dependent synergism, which was then experimentally verified. Our study demonstrates that the hierarchical mechanism of multi-site/type ubiquitination of RIG-I provides an efficient, robust and optimal synergistic regulatory module in the type I IFN signaling pathway during antiviral immune responses.

## Results

### K63-linked ubiquitination contributes greatly to RIG-I oligomerization and activation of the type I IFN signaling pathway

**Figure 1:**
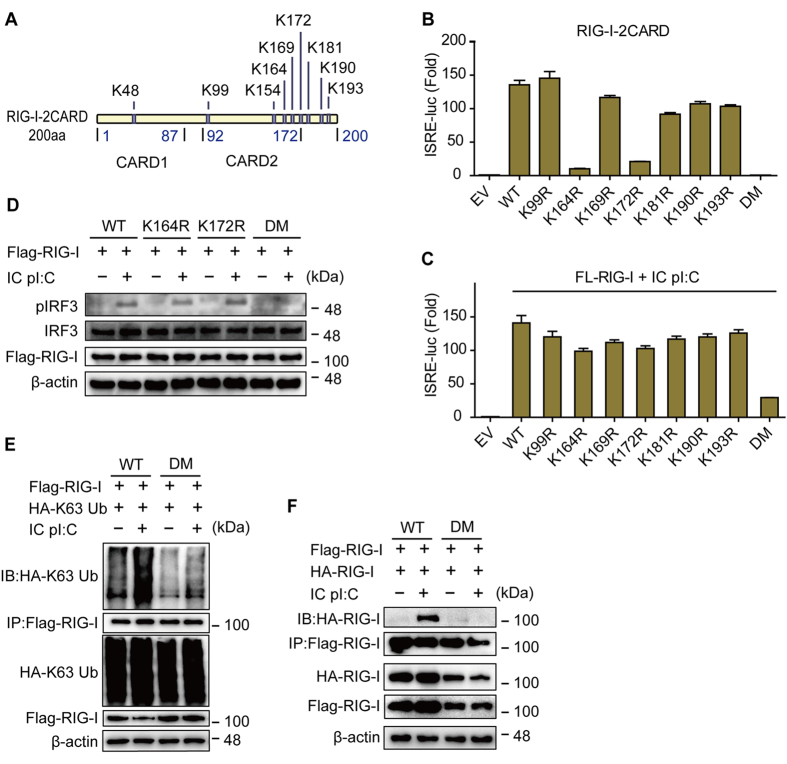
The double mutant of RIG-I markedly blocks the K63-linked ubiquitination of FL-RIG-I, whereas single mutants exhibit only small effects. (**A**) Schematic of RIG-I-2CARD ubiquitination sites. (**B**) Luciferase assay of RIG-I-2CARD and different mutants. (**C**) Luciferase assay of full length RIG-I (FL-RIG-I) or its mutants. HEK 293T cells were transfected with wild type (WT) FL-RIG-I and its mutants, together with ISRE-luc, followed by intracellular (IC) poly (I:C) treatment for 24 hr. (**D**) HEK 293T cells were transfected with WT RIG-I or its indicated mutants, followed by IC poly (I:C) treatment for 24 hr. Cell lysates were collected for immunoblot analysis. (**E**) The RIG-I K172R/K164R double mutant (DM) markedly blocks K63-linked ubiquitination of RIG-I. HEK-293T cells transfected with wild type (WT) RIG-I or DM together with HA-K63-Ub were treated with or without IC poly (I:C) for 12 hr. Lysates were collected and immunoprecipitated with anti-Flag and immunoblotted with anti-HA. (**F**) HEK-293T cells were transfected with Flag/HA-RIG-I WT or DM. After treatment with IC poly (I:C) for 9 hr, the lysates were collected and immunoprecipitated with anti-Flag and immunoblotted with anti-HA.

RIG-I ubiquitination can occur at multiple sites^7^,^11^,^12^ (Fig. 1A). The results of the detection of the function of RIG-I-2CARD mutants at most of these sites using a luciferase assay confirmed previous reports^7^,^11^. Both the K164R and K172R mutants of RIG-I-2CARD failed to initiate ISRE activation (Fig. 1B). Unexpectedly, in contrast to RIG-I-2CARD, the K164R or K172R single mutants of FL-RIG-I only partially reduced ISRE activation (Fig. 1C), whereas FL-RIG-I double mutation with K164R and K172R (DM) significantly blocked RIG-I-induced ISRE activation after intracellular (IC) poly (I:C)-LMW (a synthetic analog of double-stranded RNA with a low molecular weight has an average size of 0.2-1kb, which can only be recognized by RIG-I but not MDA5) stimulation (5 μg/mL, the same with other experiments shown below) (Fig. 1C). Detection of phosphorylated IRF3 by immunoblotting confirmed the differences in the effects of these mutations in RIG-I-2CARD and FL-RIG-I (Fig. 1D). Moreover, detection of ubiquitination levels (Fig. 1E) and the oligomerization (Fig. 1F) of wild type (WT) RIG-I and DM revealed that the ubiquitin conjugation and self-interaction of FL-RIG-I DM were also markedly impaired. These results imply that the relationship between ubiquitination of FL-RIG-I and its function may be more complicated than that of RIG-I-2CARD. There might be a precise mechanism underlying ubiquitination of FL-RIG-I, that enables robust activation of the type I IFN signaling pathway.

### Candidate models for plausible mechanisms of RIG-I ubiquitination

**Figure 2:**
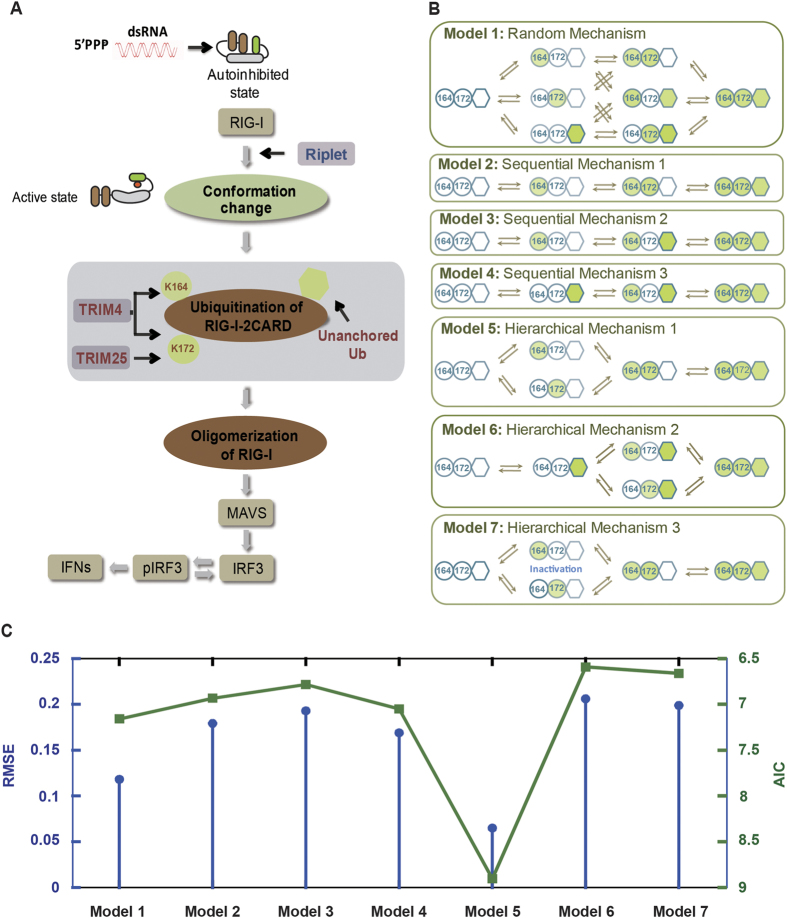
Various mechanisms of dual site/dual type ubiquitination of RIG-I and model selection. (**A**) Signaling pathway of RIG-I-induced IFN expression with a core module of multi-sites/type ubiquitination of RIG-I in antiviral response. (**B**) We proposed 7 hypothetical mechanisms with different orders of ubiquitin site/type processing of RIG-I ubiquitination (conjected ubiquitination at K164 or K172 [green circle], and unanchored ubiquitin chains binding [green hexagon]). See details in the main text. (**C**) Model selection by fitting 7 models to the experimental data. Root-mean-squared error (RMSE) and Akaike Information Criterion (AIC) were used as criteria to evaluate and select the model that best accounted for the experimental data.

Combining with the above experiments, we constructed a conceptual model for the RIG-I-induced IFN signaling pathway with a core module of RIG-I ubiquitination, as shown in Fig. 2A. The ordered mechanism in multisite protein modification^7^,^11^ is an important mechanism for regulating protein functions^15^,^16^,^17^. To discriminate different mechanisms with respect to the order of RIG-I ubiquitination at K164 and K172 as well as unanchored ubiquitin chain binding, we analyzed 7 candidate models (Fig. 2B) (conjugated ubiquitination at K164 or K172 sites and unanchored ubiquitin chain binding).

Model 1 (random mechanism) assumed that both the type and ubiquitination sites of RIG-I follow a completely random order. Models 2–4 (sequential mechanisms 1–3) assumed that RIG-I ubiquitination at K164 and K172 and the linkage of unanchored ubiquitin chains (unanchored Ub) occur in a sequential manner (i.e., K164 → K172 → unanchored Ub; K164 → unanchored Ub → K172; or unanchored Ub → K164 → K172, respectively). For simplicity, we assumed that the reactions at K164 and K172 sites were symmetric, and thus we did not explicitly consider the 3 other types of sequential mechanisms (i.e., K172 → K164 → unanchored Ub; K172 → unanchored Ub → K164; and unanchored Ub → K172 → K164). Models 5–7 (hierarchical mechanisms 1–3) distinguished the order of conjugated ubiquitination and linkage of unanchored ubiquitin chains but assumed that the ubiquitination at K164 and K172 occur at random. Specifically, model 5 (hierarchical mechanism 1) assumed that K164 and K172 are randomly ubiquitinated before linkage of unanchored ubiquitin chains, whereas model 6 (hierarchical mechanism 2) considered an opposite order. Model 7 was similar to model 5 but assumed that the activation of downstream RIG-I signaling is induced only after both K164 and K172 are ubiquitinated and that single-site ubiquitination of RIG-I does not activate downstream signaling.

### Data-driven model selection reveals a hierarchical mechanism of RIG-I ubiquitination

**Figure 3:**
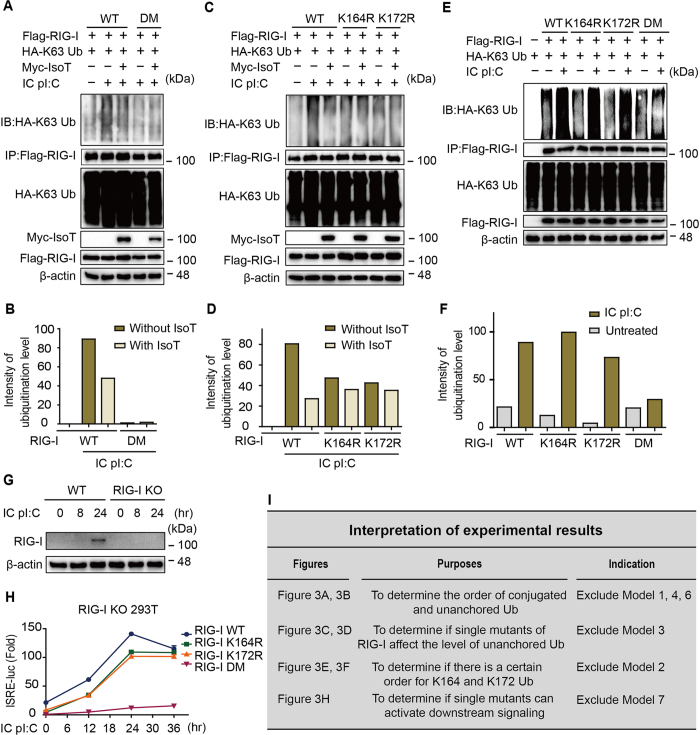
Experimental validation of the hierarchical model of RIG-I ubiquitination. (**A**) HEK-293T cells were transfected with wildtype (WT) RIG-I or the RIG-I K172R/K164R double mutant (DM) together with K63-ubiquitin (Ub) with or without IsoT, followed by intracellular (IC) poly (I:C) treatment for 12 hr. The lysates were immunoprecipitated with anti-Flag and immunoblotted with anti-HA. (**B**) Intensity quantification of RIG-I ubiquitination levels in (**A**). (**C**) HEK 293T cells were transfected with RIG-I WT, K164R or K172R together with K63-Ub, with or without IsoT, followed by IC poly (I:C) treatment for 12 hr. The lysates were immunoprecipitated with anti-Flag and immunoblotted with anti-HA. (**D**) Intensity quantification of RIG-I ubiquitination levels in (**B**). (**E**) HEK-293T cells were transfected with RIG-I WT, K164R, K172R or DM, together with K63-Ub, followed by IC poly (I:C) treatment for 12 hr. The lysates were immunoprecipitated with anti-Flag and immunoblotted with anti-HA. (**F**) Intensity quantification of RIG-I ubiquitination level in (**E**). (**G**) Knockout efficiency of RIG-I knockout (KO) HEK-293T cells. (**H)** RIG-I KO cells were transfected with FL-RIG-I or its mutants together with ISRE-luc, followed by IC poly (I:C) treatment. (**I**) Interpretation of how the experimental results support hierarchical mechanism 1 and exclude the other models.

To quantitatively examine the 7 mechanisms proposed above, we constructed a mathematical model for each mechanism according to Michaelis-Menten kinetics^18^ by employing systems of ordinary differential equations (ODEs) to model the kinetics of RIG-I ubiquitination (see details in Methods). The complete descriptions of the chemical reactions and model formulation for each mechanism are listed in Supplementary Text S1–S7. We then fitted these 7 mathematical models to our experimental data (time course and dose response) of ISRE activation levels under 4 different conditions (WT, K164R, K172R, and K164/172R; Supplementary Fig. S1). The parameters of each model were estimated by employing nonlinear least-square optimization with a condition-dependent ODE solver and genetic algorithm (Supplementary Text S8). Root-mean-squared-error (RMSE)^19^ and Akaike Information Criterion (AIC)^20^ were adopted to evaluate the goodness of fit and to select the most possible model with the greatest predictive power. As shown in Fig. 3C, model 5 (hierarchical mechanism 1) was determined to best characterize the experimental data with the lowest RMSE and AIC compared with the other models. Therefore, hierarchical mechanism 1 (i.e., K164 and K172 sites are randomly ubiquitinated followed by linkage of unanchored ubiquitin chains) might mechanistically account for RIG-I ubiquitination with the highest probability.

### Experimental validation of the hierarchical mechanism of RIG-I ubiquitination

To verify the hierarchical mechanism of RIG-I ubiquitination, we first determined the order of conjugated ubiquitination and linkage of unanchored ubiquitin chains of RIG-I. Isopeptidase T (IsoT, also named USP5) is a deubiquitinating enzyme that cleaves unanchored ubiquitin chains nonspecifically^21^ and has been widely adopted to study unanchored ubiquitination^10^,^22^. Therefore, we employed IsoT to determine the order of conjugated ubiquitination and linkage of unanchored ubiquitin chains in RIG-I. Immunoblotting (Fig. 3A,B) revealed that RIG-I DM (K164/172R) cannot link unanchored ubiquitin chains, which indicates that the linkage of unanchored ubiquitin chains to RIG-I is dependent on the ligation of anchored ubiquitin chains. These data excluded model 1 (random mechanism), model 4 (sequential mechanism 3) and model 6 (Hierarchical mechanism 2; see illustration in Fig. 3I). To determine if single mutants of RIG-I (K164R or K172R) affected the level of the linkage of unanchored ubiquitin chains, we conducted similar experiments. The experimental data (Fig. 3C,D) demonstrate that a single mutation of RIG-I also blocked the linkage of unanchored ubiquitin chains. We therefore rejected model 3 (sequential mechanism 2) from our candidate models (Fig. 3I). Furthermore, we detected the K63-linked ubiquitination level of WT RIG-I and its mutants under IC poly (I:C) stimulation (Fig. 3E). DM significantly blocked RIG-I ubiquitination, whereas K164R or K172R did not, indicating that there is no sequential order for K164 or K172 ubiquitination (Fig. 3E,F). Thus, model 2 (sequential mechanism 1) was ruled out (Fig. 3I). Hence, model 5 (hierarchical mechanism 1) and model 7 (hierarchical mechanism 3) remained. To clarify whether the single mutants could activate the signaling pathway, we generated an RIG-I knockout (KO) 293T cell line (Fig. 3G) and reconstructed the RIG-I pathway by transfecting plasmids encoding WT RIG-I or mutants (Fig. 3H). In contrast to RIG-I-DM, the RIG-I single mutants (K172R or K164R) still responded to IC poly (I:C) stimulation to activate type I IFN signaling (Fig. 3H). This result indicates that the single mutants are capable of activating the type I IFN signaling pathway (Fig. 3I). Taken together, these experiments support hierarchical mechanism 1 (model 5); that is, RIG-I ubiquitination proceeds in a hierarchical manner, as shown in Fig. 2B.

**Figure 4:**
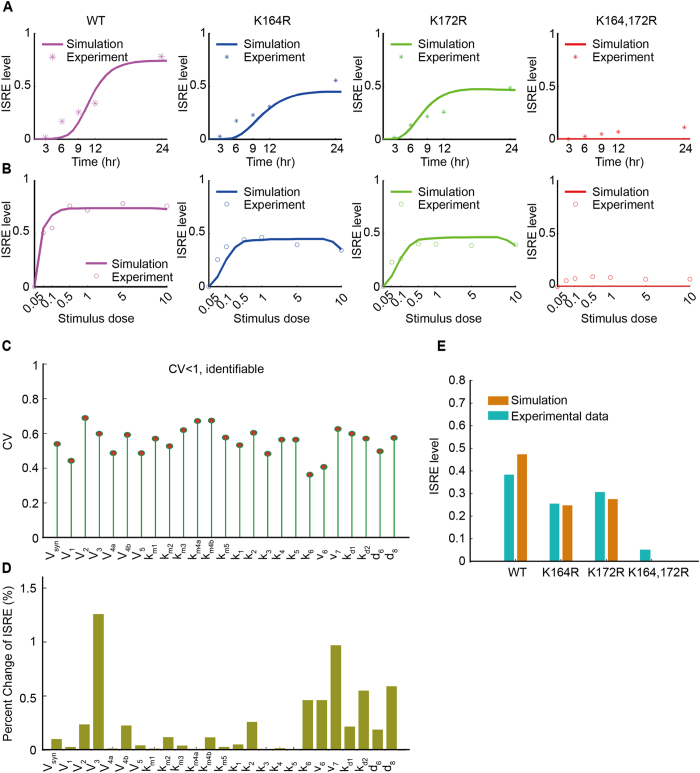
Testing the effectiveness of the selected model 5 (hierarchical mechanism 1). (**A**) Simulated (solid lines) and experimental data (dots) for ISRE activation with stimulation by intracellular (IC) poly (I:C). (**B**) Simulated and experimental data for the dose-responses of ISRE activation by IC poly (I:C) at various dosages (ranging from 0 μg/mL to 10 μg/mL). (**C**) Cross validation of the estimated parameters using the bootstrap approach. The coefficient of variation (CV) was used to evaluate the identifiability of the estimated parameters. (**D**) Parameter sensitivity analysis. (**E**) Testing the parameterized model using an independent set of luciferase assay data.

We further evaluated the predictive performance of model 5. Figure 4A,B compare the simulations from the mathematical model of hierarchical mechanism 1 and the experimental data. These results demonstrated that the prediction from our selected model is in good agreement with the experimental data. To evaluate the identifiability of the estimated parameters, we also performed leave-one-out cross validation^23^,^24^ using the bootstrap approach^25^. The coefficient of variation (CV) for each parameter was less than 1, indicating that all the parameters in the selected model were identifiable^26^,^27^ (Fig. 4C). Parameter sensitivity analysis demonstrated that the model was robust with respect to small variations in the estimated parameters (Supplementary Text S9, Fig. 4D). In addition, an independent set of luciferase assay data of ISRE was used to verify the effectiveness of the predictive model (Fig. 4E).

### Hierarchical mechanism 1 has superior performance as a compromise between sensitivity and robustness during the immune response

Our mathematical modeling and experimental validation supported a hierarchical mechanism (i.e., hierarchical mechanism 1 in the following text if not specified) of RIG-I ubiquitination. We then determined the functional role of such a hierarchical architecture in the RIG-I ubiquitination process. We hypothesized that the hierarchical architecture is superior to other mechanisms for enabling both the functional sensitivity and robustness of antiviral immune responses.

**Figure 5:**
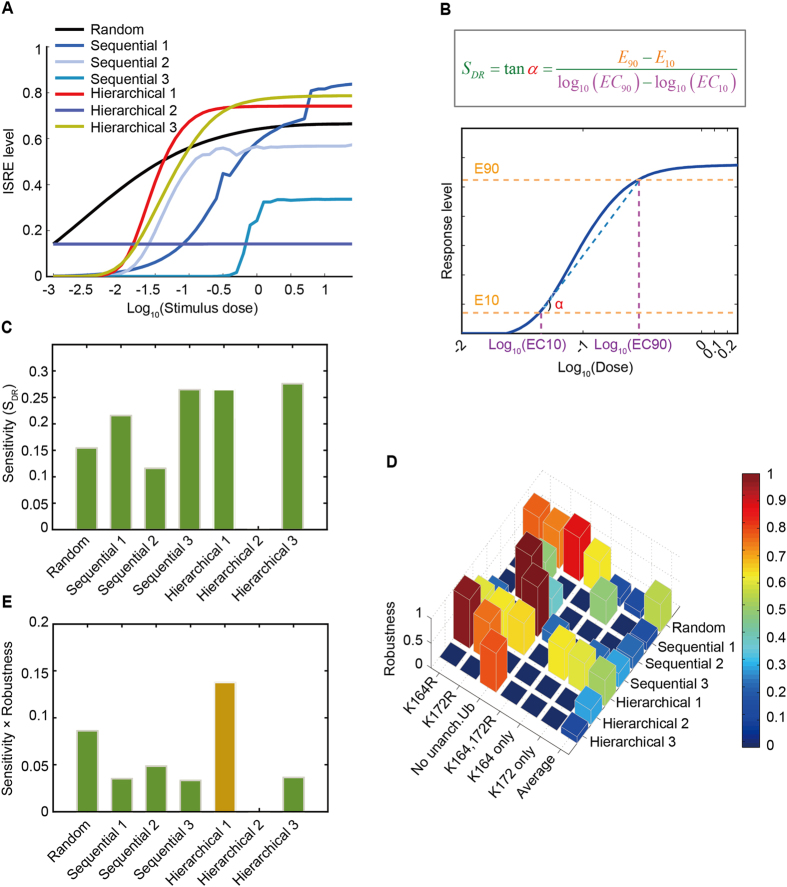
Comparisons of dose-response sensitivity and robustness produced by different mechanisms. (**A**) Dose-response curves produced by the 7 mechanisms. (**B**) Illustration of the definition of dose-response sensitivity. (**C**) Dose-response sensitivity for the 7 mechanisms. (**D**) Robustness of the 7 models to various perturbations (K164R; K172R; no unanchored ubiquitin chains [Ub], K164 and 172R; K164 only; K172 only). For each model, the average robustness with respect to 6 perturbations is also shown. See detailed results in Supplementary Table S1. (**E**) The product of sensitivity and robustness. Hierarchical mechanism 1 exhibited the greatest value of the product of sensitivity and robustness.

The dose-response curves produced by the 7 mechanisms revealed differences in the amplification of ISRE activity and its sensitivity to changes in stimuli dose in the 7 models (Fig. 5A). Inspired by the Hill coefficient^28^,^29^, we defined a “dose-response sensitivity” index (Fig. 5B) to quantify the above differences (see also Methods). Figure 5C shows the differential values of the dose-response sensitivities for the 7 mechanisms. Hierarchical mechanism 1, hierarchical mechanism 3 and sequential mechanism 3 possessed the highest dose-response sensitivities.

We then examined the robustness of RIG-I-induced ISRE activation under 6 perturbations: K164R; K172R; no unanchored poly-ubiquitin chains; K164 and172R; K164 only (i.e. no K172 and unanchored ubiquitin chains); and K172 only (i.e., no K164 and unanchored ubiquitin chains). A robustness index (see Methods) was also defined to quantify the relative changes of ISRE levels with respect to above perturbations (Supplementary Table S1). As shown in Fig. 5D, the random mechanism and hierarchical mechanism 1 possessed the greatest robustness.

We thus determined that the hierarchical mechanism 1 exhibits good performance in both sensitivity and robustness during antiviral responses, whereas the other models are either less sensitive or less robust (Fig. 5E). Therefore, as a compromise between sensitivity and robustness^30^,^31^, the hierarchical mechanism is superior to other mechanisms.

### Optimal modulation of synergism between TRIM4 and TRIM25 during antiviral responses

TRIM4 and TRIM25 are critical in modulating type I IFN induction by targeting RIG-I at the K164 and K172 sites for K63-linked ubiquitination^7^,^11^. Our model simulation (Supplementary Fig. S3) also demonstrated tunability of antiviral responses by manipulating dual-site ubiquitination of RIG-I with the combination of TRIM4 and TRIM25.

**Figure 6:**
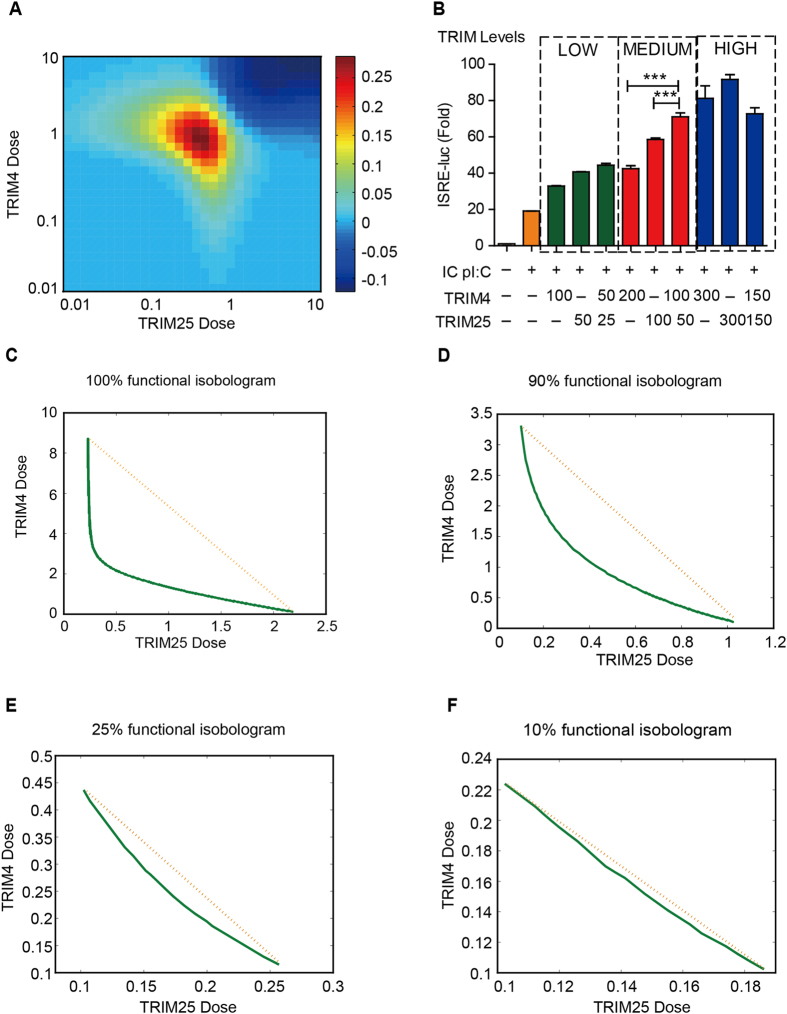
Optimal modulation of synergism between TRIM4 and TRIM25. (**A**) The dose-dependent synergism of the combination of TRIM4 and TRIM25 was evaluated using Bliss combination index. The maximal synergy was achieved in the region of medium doses of TRIM4 and TRIM25. As the dose of TRIMs became high, the pattern switched from synergism to antagonism. (**B**) Experimental validation of the dose-dependent synergism between TRIM4 and TRIM25. Three sets of luciferase assays evaluated ISRE levels under treatments of single TRIM4, single TRIM25 and combined TRIM4 and TRIM25 at 3 different dosages (low, medium and high). (**C**–**F**) Isobologram synergism analysis for TRIM4 and TRIM25 during immune responses. The Loewe Index was used to evaluate the combinatorial effect of TRIM4 and TRIM25. If *x*% of the isobologram of TRIM4 and TRIM25 (green curve) bowed inward (indicating *CI*_*Loewe*_ < 1), then the combination of TRIM4 and TRIM25 had synergistic effect. (**C**) 100%, (**D**) 90%, (**E**) 25% and (**F**) 10% function isobologram analyses were performed.

We examined whether TRIM4 and TRIM25 function cooperatively in antiviral responses by employing the Bliss combination index^32^ (see Methods) to quantitatively evaluate the synergism between TRIM4 and TRIM25. Figure 6A reveals a dose-dependent synergism of this combination. As the doses (or concentrations) of combined TRIM4 and TRIM25 increased, the synergism pattern switched from synergy (*CI*_*Bliss*_ > 1) to antagonism (*CI*_*Bliss*_ < 1). Maximal synergy was achieved in a region of medium doses of TRIM4 and TRIM25. Experiments with a luciferase assay validated the predicted combinatorial effects of TRIM4 and TRIM25 (Fig. 6B). An obviously synergistic effect was observed at intermediate levels of TRIM4 and TRIM25. Moreover, our experimental data confirmed that low or high doses of combinatorial TRIM4 and TRIM25 eliminated obvious synergistic effects or produced antagonistic effects. These experimental results agreed well with our model prediction.

Finally, we analyzed the isobologram of antiviral responses with respect to TRIM4 and TRIM25 by adopting the Loewe combination index^32^ (see Methods); 100%, 90%, 25% and 10% isobolograms (Fig. 6C,F) were evaluated. The model predicted that TRIM4 and TRIM25 exhibit differential synergies for different objectives. Specifically, TRIM4 and TRIM25 function in synergy to achieve a high objective of functional immune responses (100 and 90% function, Fig. 6C,D), but function in slight synergy or near additivity to achieve a low objective of functional responses (25 and 10% function, Fig. 6E,F). This objective-dependent synergism characterizes an optimal and economic cooperative regulatory system in innate immune responses.

## Discussion

This study employed a systems biology approach to explore the biochemical reaction mechanism underlying multi-site/type ubiquitination of RIG-I in innate immune responses. Data-driven model selection and experimental validation revealed a novel hierarchical mechanism of RIG-I ubiquitination. Compared with random and sequential mechanisms, this hierarchical mechanism performed better with respect to robustness and sensitivity during antiviral responses. In addition, our study demonstrated an optimal synergism between TRIM4 and TRIM25 in promoting RIG-I ubiquitination and activating the IFN signaling pathway.

Type I IFNs, a paradigm of transcriptional regulation, have been studied for many years in antiviral responses. RIG-I plays an indispensable role in recognizing short dsRNA from infected viruses^33^. Ubiquitination is one of the most important modifications for the complex activation of RIG-I. Based on reported K63-linked ubiquitin sites, we compared functions of mutations of these sites in RIG-I-2CARD and FL-RIG-I and observed differential responses between the two constructs, possibly due to different protein conformations. Moreover, among the many ubiquitination sites, we determined that K164 and K172 are essential for robust RIG-I K63-linked ubiquitination because ubiquitination of RIG-I and its function were significantly impaired only when both of these two ubiquitination sites were mutated.

In addition to the conjugated poly-ubiquitin chains, unanchored K63-linked ubiquitin chains are required to activate RIG-I^10^. To determine the order of RIG-I conjugated ubiquitination and unanchored ubiquitin chain linkages, we conceived several possible models. By fitting the corresponding mathematical model to the experimental data, we identified model 5 (hierarchical mechanism 1) as a mechanistic model for RIG-I ubiquitination, which was then experimentally verified by co-immunoprecipitation, *in vivo* ubiquitination assays and luciferase assays. Specifically, K63-linked ubiquitin chains conjugate to the most important ubiquitin sites, K164 and K172, in random order, whereas the interaction between RIG-I and unanchored ubiquitin chains occurs after conjugated ubiquitination at both sites.

Many experimental studies have demonstrated that the innate immune response system is precisely and robustly regulated^34^. Conserving sensitivity and robustness are thus important “design principles”^35^ of such a system. However, there is always a trade-off between sensitivity and robustness in a dynamic system^30^,^36^. Different architectures of biological networks may have different performances in sensitivity and robustness. As demonstrated in this study, RIG-I ubiquitination via a random mechanism is rather robust to various perturbations but is not sensitive to changes in the strength of stimuli. By contrast, in a sequential mechanism (including sequential mechanisms 1 and 2) and hierarchical mechanism 3, RIG-I ubiquitination and IFN expression are sensitive to changes in the strength of stimuli but fragile with respect to the perturbations of ubiquitin sites. By contrast, hierarchical mechanism 1 allows RIG-I-mediated type I IFN induction to be both robust and sensitive. By comparing different models, the superiority sensitivity and robustness of the hierarchical mechanism provides insights on the functional role of the architecture in RIG-I ubiquitination processing. In addition, the hierarchical mechanism might provide an additional framework for further theoretical studies on system design.

Since Gack *et al.* reported that K63 ubiquitin chains generated by TRIM25 play a crucial role in activation of RIG-I-induced type I IFN signaling^37^, studies have increasingly focused on the molecular mechanisms underlying the regulation of RIG-I ubiquitination. Several positive and negative regulators of RIG-I ubiquitination have been identified. For instance, CYLD, USP3 and USP21 inhibit the type I IFN signaling pathway by removing K63-linked ubiquitin chains from RIG-I^38^,^39^,^40^, whereas TRIM4, TRIM25 and Riplet play positive roles^13^,^37^,^41^. However, it is unclear if and how multiple molecules acting on the same adaptor cooperate with each other to optimally regulate the immune response. In this study, we determined that ISRE activation was flexibly modulated by manipulating the ubiquitination of RIG-I at K164 and K172 sites via the combination of TRIM4 and TRIM25. Furthermore, quantitative model analysis and experimental verification demonstrated that TRIM4 and TRIM25 exhibit synergism in a dose/objective-dependent manner (Fig. 6). These results might unravel the salient features of optimal modulation in RIG-I ubiquitination against viral infection. The dose-dependent synergism of TRIM4 and TRIM25 might also enhance the robust performance of immune responses in noisy environments.

We also examined which component(s) of the RIG-I signaling system ensure the existence of synergy between TRIM4 and TRIM25 during the innate immune response. We noted that the dose-dependent synergism (Fig. 6A) might be a consequence of nonlinear regulation and responses. We therefore examined the component with nonlinearity in the model of the RIG-I mediated type I IFN signaling pathway. We hypothesized that oligomerization of RIG-I, which possesses strong nonlinearity, might contribute to this synergy. Therefore, we examined the model without oligomerization of RIG-I (by setting the corresponding power of the RIG-I tetramer, 4. to 1 in the mathematical model) and evaluated the synergism between TRIM4 and TRIM25 using the Bliss combination index (Supplementary Fig. S4). Compared to the WT model (with oligomerization of RIG-I; Fig. 6A), the loss of oligomerization of RIG-I resulted in reduced synergy between TRIM4 and TRIM25. This analysis indicated an essential role of hierarchical ubiquitination-based oligomerization of RIG-I in producing synergy during antiviral immune responses, which is also consistent with previous studies^42^.

In summary, using an integrated experimental and modeling approach, we have revealed a hierarchical mechanism for RIG-I ubiquitination in which conjugated K63-linked ubiquitination at two lysine sites precedes unanchored ubiquitin chain linkage. This hierarchical architecture ensures that RIG-I-induced type I IFN signaling activation is both sensitive and robust in antiviral immune responses. Furthermore, our study demonstrates that the activation of type I IFN signaling is synergistically modulated by two E3-ubiquitin ligases (TRIM4 and TRIM25) that act at dual sites (K164, K172) of RIG-I in an optimal manner. This study advances our understanding of the molecular mechanism and design principles for multi-site/type ubiquitination of RIG-I as well as its physiological functions in innate immune responses.

## Methods

### Cell culture and reagents

Human embryonic kidney 293T (HEK-293T) cells were cultivated in DMEM (Hyclone) supplemented with 10% fetal bovine serum (Gibco) and 1% L-glutamine (Gibco) at 37 °C in 5% CO_2_. Overexpression plasmids were transfected using Lipofectamine 2000 reagent (Invitrogen) according to the manufacturer’s instructions. Poly (I:C) (LMW) was purchased from Invivogen.

### Luciferase reporter assays

HEK-293T (5×10^4^) cells were planted in 24-well plates and transfected with plasmids encoding the ISRE luciferase reporter (firefly luciferase; 20 ng) and pRL-TK (Renilla luciferase plasmid; 8 ng) together with Flag-RIG-I WT and the different mutants (100 ng). Cells were harvested after intracellular poly (I:C) (LMW) stimulation for the indicated times in passive lysis buffer (Promega). Enzyme activity was normalized by the efficiency of transfection on the basis of Renilla luciferase activity levels, and the results are presented as the fold induction relative to the basal level measured in cells. The values are the mean ± SD of three independent transfections performed in parallel.

### Immunoprecipitation and immunoblot analysis

Cells were extracted in ice-cold low-salt lysis buffer (50 mM Hepes pH 7.5, 150 mM NaCl, 1 mM EDTA, 1.5 mM MgCl_2_, 10% glycerol, 1% Triton X-100) supplemented with 5 mg/mL protease inhibitor cocktail (Roche). A 20-μL aliquot of each sample was subjected to SDS-PAGE. For immunoprecipitation (IP) experiments, whole-cell extracts were incubated with anti-Flag agarose gels (Sigma) overnight. The beads were washed three times with low-salt lysis buffer. The immunoprecipitates were resuspended in 3×SDS Loading Buffer (FD Biotechnology) and boiled for 5 minutes. The released proteins were electrophoresed on 8–12% SDS-polyacrylamide gels and transferred onto PVDF membranes, with subsequent blocking using 5% skim milk. The membranes were incubated with the indicated antibodies and detected using enhanced chemiluminescence (Millipore).

### Knockout of RIG-I by the CRISPR Cas9 system

We analyzed gRNA in the website http://crispr.mit.edu/ and chose the gRNA sequence with the highest score to design primers:

primer1: AATACTGTTTTCTTTTTGAAAAATCAA;

primer2: AAGTACTATTCTATGAGTACTTTTGT.

Primers were annealed and then linked to the pCRISPR-V2 vector.

### Mathematical modeling

As depicted in Fig. 2A, during viral (e.g., dsRNA) infection, Riplet promotes the K63-linked ubiquitination of the RIG-I C-terminal domain, which results in a conformational change^13^ of RIG-I and subsequent exposure of the CARD domain. RIG-I can be ubiquitinated at K164 by the E3 ligase TRIM4^11^ or at K172 by TRIM25 or TRIM4^7^,^11^. In addition to these conjugated ubiquitin chains, RIG-I interacts with unanchored ubiquitin chains^10^. RIG-I can be de-ubiquitinated by various deubiquitinating enzymes (e.g., USP3 and USP21)^38^,^39^.

We hypothesized 7 mechanisms for dual-site/dual-type ubiquitination of RIG-I (Fig. 2B) and constructed the corresponding mathematical models for each mechanism by employing ordinary differential equations (ODEs) to describe dynamic ubiquitination of RIG-I according to Michaelis-Menten kinetics^18^,^43^. A complete description of the biochemical reactions, rate equations and ODEs to model 7 mechanisms are listed in Supplementary Text S1–S7. Below we only describe the mathematical model for hierarchical mechanism 1 (Model 5).

Hierarchical mechanism 1 (Model 5) assumes that K164 and K172 are randomly ubiquitinated before unanchored ubiquitination occurs (see biochemical reactions and rate equation in Supplementary Text S5). The corresponding ODE model is described by the following equations:


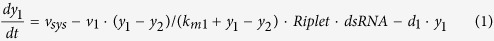



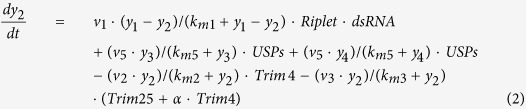



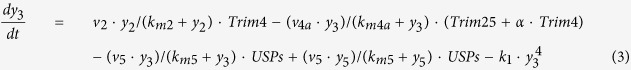



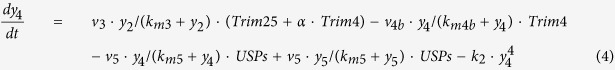



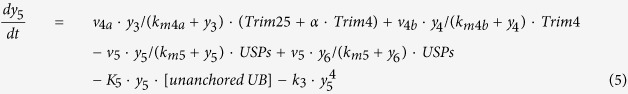



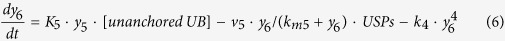


where *y*_1_ represents the expression level of RIG-I, *y*_2_ represents Riplet-ubiquitinated RIG-I with a conformational change, *y*_3_ and *y*_4_ represent the ubiquitination of RIG-I at K164 and K172 respectively, whereas *y*_5_ represents the ubiquitination of RIG-I at both K164 and K172, and *y*_6_ represents further ubiquitinated RIG-I binding with unanchored ubiquitin chains. The biological meanings of the parameters involved in the model are listed in Supplementary Tables S2–S3.

Ubiquitination of RIG-I at K164 or K172 can induce various RIG-I tetramers^44^. Promoted by unanchored ubiquitin chains, RIG-I that has been ubiquitinated by TRIMs can also form the tetramer^8^, which is reversibly balanced by the action of deubiquitinating enzymes (i.e. USPs)^45^,^46^. The RIG-I tetramer degrades rapidly after activating the downstream molecules. These processes are described by the following equation:


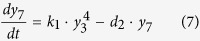



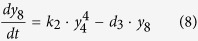



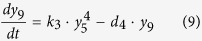



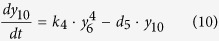


where *y*_7_, *y*_8_ and *y*_9_ represent tetramers of RIG-I that have been ubiquitinated at K164, K172, or both sites, respectively, and *y*_*10*_represents the tetramer of fully ubiquitinated RIG-I promoted by unanchored ubiquitin chains.

RIG-I tetramer interacts with MAVS and then phosphorylates IRF3. Phosphorylated IRF3 can activate ISRE, a promoter of the IFN gene. To integrate less critical reaction details, we employed Hill functions^47^ to model the regulations involved in the signaling pathway of pIRF3/ISRE/IFNs, which are described by the following equations:


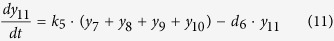



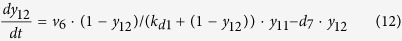



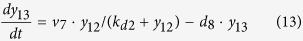


where *y*_11_, *y*_12_ and *y*_13_ represent the activation levels of MAVS, phosphorylated IRF3 and activated ISRE, respectively.

### Parameter estimation

Nonlinear least-square optimization was employed to estimate the unknown parameters in the ODE model by minimizing the fitness error between the simulation results and the experimental data. Two types of experimental data, including the time-course and dose-response of ISRE under 4 different conditions (WT, K164R, K172R and K164&172R; Supplementary Fig. S1), were integrated into the fitting using the following objective function:


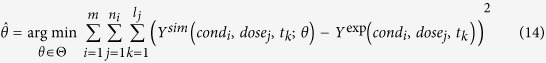


where 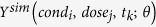 represents the model-simulated ISRE level at time point *t*_*k*_ under the *i-*th condition (*cond*_*i*_) and *j*-th dose of stimulus (*dose*_*j*_) with parameter set *θ*; 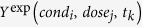 is the corresponding experimental data; and Θ represents the parameter space.

The degradation rates of RIG-I and CARD as well as the dephosphorylation rate of pIRF3 were measured experimentally (Supplementary Fig. S2) to reduce the number of unknown parameters. To fit unknown parameters in the model to the condition-dependent time course data and dose-response data simultaneously, we designed a conditions-dependent 4^th^ Runge-Kutta method to solve the system of ODEs, associated with a modified genetic algorithm^48^. Multiple sets of starting values of parameters were sampled to repeat the estimation in the genetic algorithm. The detailed optimization algorithm used to estimate the parameters is described in Supplementary Text S8. The estimated parameter values are listed in Supplementary Table S2. According to the experiments, the normalized initial value of *y*_1_ was set as 1, and the initial values of other variables were assumed to be 0.

### Criterion for model selection

Root-mean-square error (RMSE)^19^ and Akaike Information Criterion (AIC)^20^ were used to evaluate and select the model that best accounted for the experimental data. The RMSE was defined as follows:


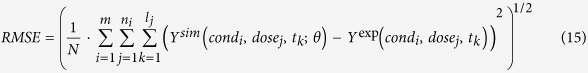


where 
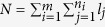
 is the number of data points. 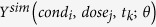 and 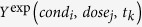 were described above.

The AIC value quantifies the trade-off between the goodness of fit and the complexity of the model to avoid selecting a model that is too complex. For a model with *p* parameters fitted to the experimental data with *N* data points, AIC is calculated as follows:


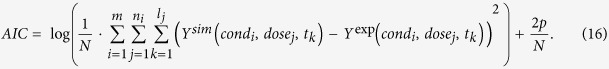


Given a set of candidate models with different complexities and thus different parameter numbers, the lowest RMSE and AIC suggest the preferred model that possesses both good predictive power and appropriate simplicity.

### Dose-response sensitivity

The Hill coefficient^28^,^29^ is usually used to quantitatively evaluate the ultrasensitivity of a dose-response curve (in the sense that it is “more sensitive” than a Michaelis-Menten response to stimulus). However, the Hill coefficient does not consider differential response levels and hence falls short in evaluating the differential amplification of responses produced by different mechanisms. Therefore, inspired by the Hill coefficient, we designed a “dose-response sensitivity” index (Fig. 4B) to describe both the amplification and sensitivity of immune responses to dose changes in virus stimuli as follows:


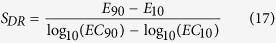


where *E*_90_ and *E*_10_ are 90 and 10% of the maximal response and *EC*_90_ and *EC*_10_ represent the strengths of stimulus that generate 90 and 10% of the maximal response, respectively.

### Robustness

We examined the robustness of the RIG-I mediated IFN signaling pathway to a stimulus (viral dsRNA treatment) under various perturbations: K164R; K172R; no unanchored ubiquitin chains; K164, 172R; K164 only (i.e. no K172 and no unanchored ubiquitin chains); K172 only (i.e., no K164 and no unanchored ubiquitin chains). Under certain perturbations, if ISRE activation was maintained at an effective level, we considered the system robust with respect to this perturbation. Hence, we defined the following index to quantitatively evaluate the robustness of the system to the above perturbations:


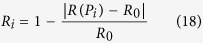


where *R*(*P*_*i*_) is the ISRE response level after 24 hours under perturbation *P*_*i*_ and *R*_0_ is the ISRE response level without perturbation. The second term evaluates the relative change in the ISRE response. Therefore, under perturbation *P*_*i*_, a smaller relative change in ISRE corresponds to greater robustness *R*_*i*_.

### Synergy evaluation

We used the Bliss combination index^49^,^50^ to quantitatively examine whether TRIM4 and TRIM25 function cooperatively or synergistically during anti-virus immune responses. This index is defined by the following equation:


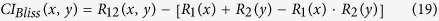


where *R*_1_(*x*), *R*_2_(*y*) and *R*_12_(*x*, *y*) are the relative response levels of ISRE to TRIM4 (at a dose of *x*), TRIM25 (at a dose of *y*) and their combination (at doses of [*x*, *y*]), respectively. Therefore, *CI*_*Bliss*_ > 0, *CI*_*Bliss*_ < 0 and *CI*_*Bliss*_ = 0 indicate synergistic, antagonistic and additive combination effects, respectively.

Furthermore, we employed the Loewe combination index^32^,^50^,^51^ to examine whether TRIM4 and TRIM25 function synergistically for a given response objective. This index is defined as follows:


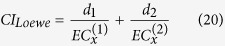


where *d*_*1*_ and *d*_*2*_ are the concentrations of combinatory TRIM4 and TRIM25 in the combination isobologram with respect to the *x* percentage of the maximal ISRE level. 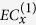 and 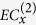 represent the concentrations of single TRIM4 and TRIM25 with respect to promoting ISRE activation by *x* percentage, respectively. *CI*_*Loewe*_ < 1, *CI*_*Loewe*_ > 1 and *CI*_*Loewe*_ = 1 indicate synergy, antagonism, and additivity, respectively.

## Additional Information

**How to cite this article**: Sun, X. *et al.* A Hierarchical Mechanism of RIG-I Ubiquitination Provides Sensitivity, Robustness and Synergy in Antiviral Immune Responses. *Sci. Rep.* **6**, 29263; doi: 10.1038/srep29263 (2016).

## Supplementary Material

Supplementary Information
