# Supplementary material for: A Hierarchical Mechanism of RIG-I Ubiquitination Provides Sensitivity, Robustness and Synergy in Antiviral Immune Responses
Source: Sci Rep. 2016 Jul 8;6:29263. doi: 10.1038/srep29263 (PMC4937349; doi:10.1038/srep29263)
Supplement: Supplementary Information [file srep29263-s1.pdf]

Supplementary Information for

A Hierarchical Mechanism of RIG-I Ubiquitination Provides Sensitivity, Robustness and Synergy of Antiviral Immune Response

Xiaoqiang Sun[1,2,3#], Huifang Xian[2#], Shuo Tian[2], Tingzhe Sun[4], Yunfei Qin[5], Shoutao Zhang[5*], Jun Cui[2,6*]

1 Zhong-shan School of Medicine, Sun Yat-sen University, Guangzhou 510089, China

2 School of Life Science, Sun Yat-sen University, Guangzhou, 510275, China

3 School of Mathematical and Computational Science, Sun Yat-sen University, Guangzhou, 510000, China

4 School of Life Sciences, AnQing Normal University, AnQing, 246011, China

5 School of Life Sciences, Zhengzhou University, Zhengzhou, Henan, 450001, China

6 Collaborative Innovation Center of Cancer Medicine, Sun Yat-sen University, Guangzhou, 510060, China

# These authors contributed equally to this work.

* Corresponding authors (SZ: zhangst@zzu.edu.cn, JC: cuij5@mail.sysu.edu.cn)

Figure S1 Experimental data used for parameter estimation

Figure S2 Experimentally measured degradation rates

Figure S3 Simulated dose-response surface of ISRE

Figure S4 Synergism evaluation for TRIM4 and TRIM25

Text S1 Model formulation for random mechanism

Text S2 Model formulation for sequential mechanism 1

Text S3 Model formulation for sequential mechanism 2

Text S4 Model formulation for sequential mechanism 3

Text S5 Model formulation for hierarchical mechanism 1

Text S6 Model formulation for hierarchical mechanism 2

Text S7 Model formulation for hierarchical mechanism 3

Text S8 Optimization algorithm

Text S9 Parameter sensitivity analysis

Table S1 Quantification of robustness indexes of 7 models

Table S2 Estimated parameter values

Table S3 Experimentally measured degradation rates

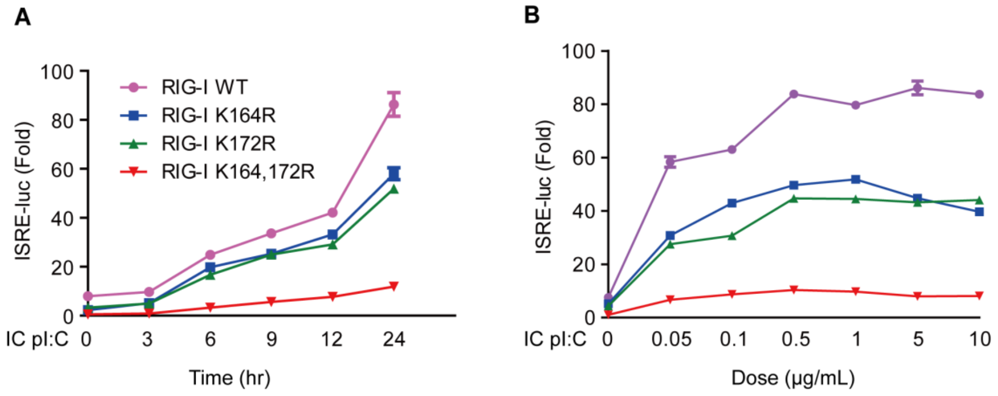

Fig. S1. Experimental data used for parameter estimation. The data includes the luciferace assay of time-course (**A**) and dose-response (**B**) of ISRE activation level under 4 different conditions (WT, K164R, K172R and k164&172R). **(A)** HEK 293T cells were treated with intracellular (IC) poly (I:C) at different time points. **(B)** HEK 293T cells were treated with the indicated doses of IC poly (I:C). Results were shown as fold induction of ISRE relative activation to the basal level measured in cells. Values are mean ± SD from three independent transfections performed in parallel.

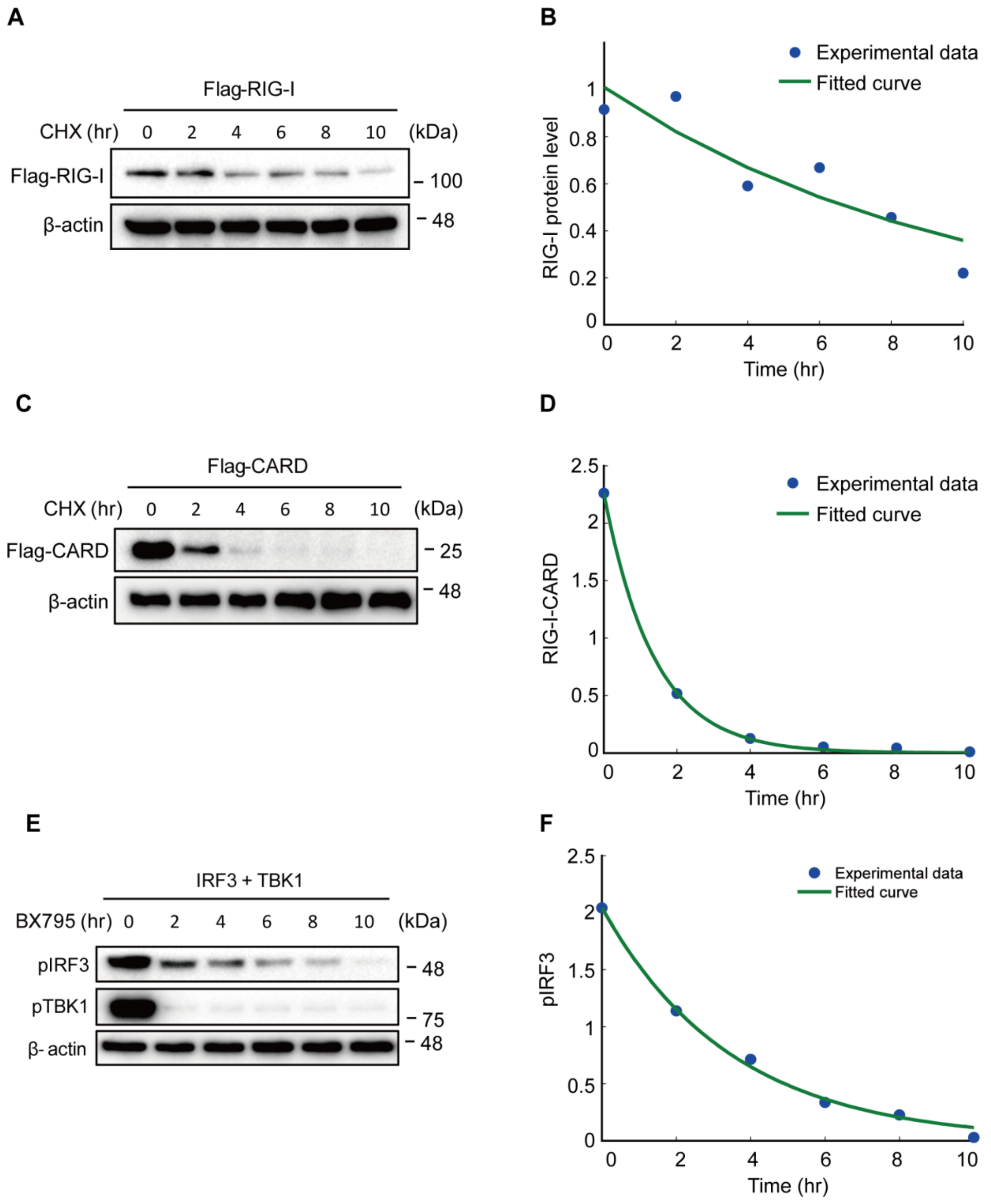

Fig. S2. Experimentally measured degradation rates. (**A-B**) RIG-I degradation rate was detected by treating with cycloheximide (CHX), a protein synthesis inhibitor. (**A**) Immunoblot for RIG-I degradation at the indicated times. (**B**) Dots are experimental results. Solid line is the best fitted curve of RIG-I degradation. The estimated degradation rate of RIG-I is 0.103534. (**C-D**) RIG-I-2CARD degradation rate. (**C**) Immunoblot blots for RIG-I-2CARD degradation at the indicated times. (**D**) Dots are experimental results. Solid line is the best fitted curve of RIG-I

degradation. The estimated degradation rate of RIG-I-2CARD is 0.729859. (**E-F**) Dephosphorylation rate of pIRF3 was detected by using BX795, a TBK1 inhibitor. (**E**) Immunoblots for the phosphorylation levels of IRF3 and TBK1 at the indicated times. (**F**) Dots are experimental results. Solid line is the best fitted curve of IRF3 dephosphorylation. The estimated dephosphorylation rate of pIRF3 is 0.28678.

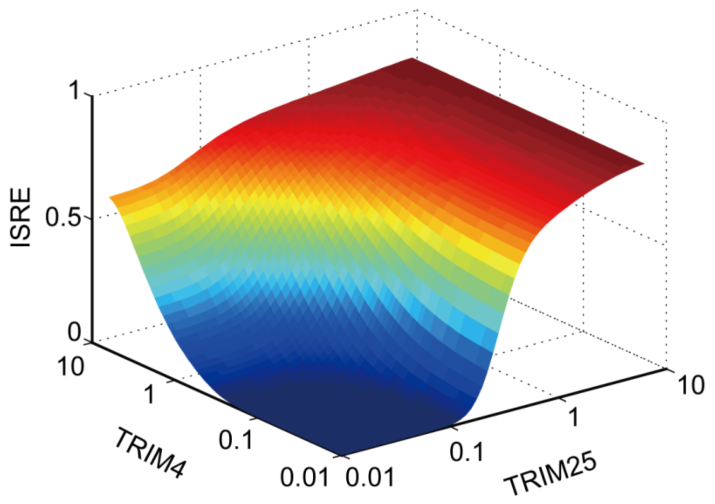

Fig. S3. Simulated dose-response surface of ISRE with respect to different dosages of combined TRIM4 and TRIM25.

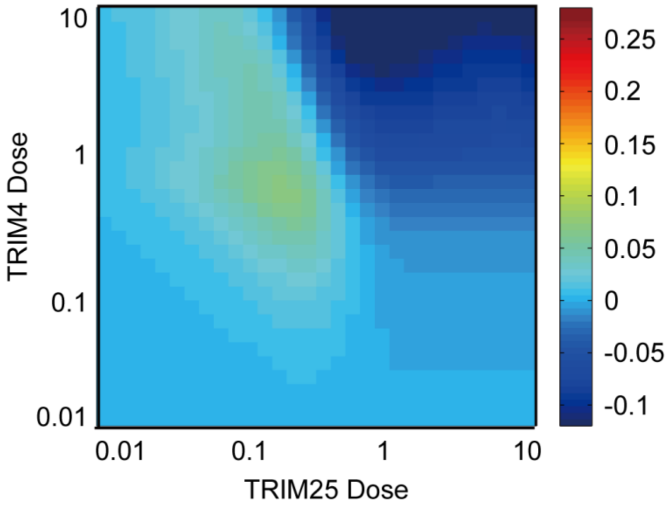

Fig. S4. Synergism evaluation for TRIM4 and TRIM25 based upon the model with no oligomerization of RIG-I. Bliss combination index was used to evaluate the dose-dependent synergism for the combination of TRIM4 and TRIM25. Compared to the wide type model (with the oligomerization of RIG-I) (Figure 7A), loss of RIG-I oligomerization resulted in reduced synergy between TRIM4 and TRIM25, indicating an essential role of hierarchical ubiquitination-based oligomerization of RIG-I in producing synergy during antiviral innate immune response.

Text S1 Model formulation for random mechanism

1.1 Biochemical reactions of RIG-I ubiquitination in random mechanism

R_1 $\quad \mathrm{RIG-I} + \mathrm{dsRNA} \xrightarrow{\mathrm{Riplet}} \mathrm{RIG-I_{uc}}$

R_2 $\quad \mathrm{RIG-I} \xrightarrow{\mathrm{Degradation}} \mathrm{null}$

R_3 $\quad \mathrm{RIG-I_{uc}} \xrightarrow{\mathrm{TRIM4}} \mathrm{ub_{164}RIG-I_{uc}}$

R_4 $\quad \mathrm{ub_{164}RIG-I_{uc}} \xrightarrow{\mathrm{USPs}} \mathrm{RIG-I_{uc}}$

R_5 $\quad \mathrm{RIG-I_{uc}} \xrightarrow{\mathrm{TRIM4,TRIM25}} \mathrm{ub_{172}RIG-I_{uc}}$

R_6 $\quad \mathrm{ub_{172}RIG-I_{uc}} \xrightarrow{\mathrm{USPs}} \mathrm{RIG-I_{uc}}$

R_7 $\quad \mathrm{RIG-I_{uc}} \xrightarrow{\mathrm{unanchored\ UB}} \mathrm{RIG-I_{uc}-unanc}$

R_8 $\quad \mathrm{RIG-I_{uc}-unanc} \xrightarrow{\mathrm{USPs}} \mathrm{RIG-I_{uc}}$

R_9 $\quad \mathrm{ub_{164}RIG-I_{uc}} \xrightarrow{\mathrm{TRIM4,TRIM25}} \mathrm{ub_{172}^{164}RIG-I_{uc}}$

R_{10} $\quad \mathrm{ub_{172}^{164}RIG-I_{uc}} \xrightarrow{\mathrm{USPs}} \mathrm{ub_{164}RIG-I_{uc}}$

R_{11} $\quad \mathrm{ub_{172}RIG-I_{uc}} \xrightarrow{\mathrm{TRIM25}} \mathrm{ub_{172}^{164}RIG-I_{uc}}$

R_{12} $\quad \mathrm{ub_{172}^{164}RIG-I_{uc}} \xrightarrow{\mathrm{USPs}} \mathrm{ub_{172}RIG-I_{uc}}$

R_{13} $\quad \mathrm{ub_{164}RIG-I_{uc}} \xrightarrow{\mathrm{unanchored\ UB}} \mathrm{ub_{164}RIG-I_{uc}-unanc}$

R_{14} $\quad \mathrm{ub_{164}RIG-I_{uc}-unanc} \xrightarrow{\mathrm{USPs}} \mathrm{ub_{164}RIG-I_{uc}}$

R_{15} $\quad \mathrm{ub_{172}RIG-I_{uc}} \xrightarrow{\mathrm{unanchored\ UB}} \mathrm{ub_{172}RIG-I_{uc}-unanc}$

R_{16} $\quad \mathrm{ub_{172}RIG-I_{uc}-unanc} \xrightarrow{\mathrm{USPs}} \mathrm{ub_{172}RIG-I_{uc}}$

R_{17} $\quad \mathrm{RIG-I_{uc}-unanc} \xrightarrow{\mathrm{TRIM4}} \mathrm{ub_{164}RIG-I_{uc}-unanc}$

R_{18} $\quad \mathrm{ub_{164}RIG-I_{uc}-unanc} \xrightarrow{\mathrm{USPs}} \mathrm{RIG-I_{uc}-unanc}$

R_{19} $\quad \mathrm{RIG-I_{uc}-unanc} \xrightarrow{\mathrm{TRIM4,TRIM25}} \mathrm{ub_{172}RIG-I_{uc}-unanc}$

R_{20} $\quad \mathrm{ub_{172}RIG-I_{uc}-unanc} \xrightarrow{\mathrm{USPs}} \mathrm{RIG-I_{uc}-unanc}$

R_{21} $$ub_{172}^{164}RIG-I_{uc} \xrightarrow{\text{unanchored UB}} ub_{172}^{164}RIG-I_{uc}-unanc$$

R_{22} $$ub_{172}^{164}RIG-I_{uc}-unanc \xrightarrow{\text{USPs}} ub_{172}^{164}RIG-I_{uc}$$

R_{23} $$ub_{164}RIG-I_{uc}-unanc \xrightarrow{\text{TRIM4,TRIM25}} ub_{172}^{164}RIG-I_{uc}-unanc$$

R_{24} $$ub_{172}^{164}RIG-I_{uc}-unanc \xrightarrow{\text{USPs}} ub_{164}RIG-I_{uc}-unanc$$

R_{25} $$ub_{172}RIG-I_{uc}-unanc \xrightarrow{\text{TRIM4}} ub_{172}^{164}RIG-I_{uc}-unanc$$

R_{26} $$ub_{172}^{164}RIG-I_{uc}-unanc \xrightarrow{\text{USPs}} ub_{164}RIG-I_{uc}-unanc$$

R_{27} $$4*ub_{164}RIG-I_{uc} \to (ub_{164}RIG-I_{uc})_4$$

R_{28} $$4*ub_{172}RIG-I_{uc} \to (ub_{172}RIG-I_{uc})_4$$

R_{29} $$4*RIG-I_{uc}-unanc \to (RIG-I_{uc}-unanc)_4$$

R_{30} $$4*ub_{172}^{164}RIG-I_{uc} \to (ub_{172}^{164}RIG-I_{uc})_4$$

R_{31} $$4*ub_{164}RIG-I_{uc}-unanc \to (ub_{164}RIG-I_{uc}-unanc)_4$$

R_{32} $$4*ub_{172}RIG-I_{uc}-unanc \to (ub_{172}RIG-I_{uc}-unanc)_4$$

R_{33} $$4*ub_{172}^{164}RIG-I_{uc}-unanc \to (ub_{172}^{164}RIG-I_{uc}-unanc)_4$$

R_{34} $$(ub_{164}RIG-I_{uc})_4 \xrightarrow{\text{Degradation}} null$$

$$(ub_{172}RIG-I_{uc})_4 \xrightarrow{\text{Degradation}} null$$

$$(RIG-I_{uc}-unanc)_4 \xrightarrow{\text{Degradation}} null$$

$$(ub_{172}^{164}RIG-I_{uc})_4 \xrightarrow{\text{Degradation}} null$$

$$(ub_{164}RIG-I_{uc}-unanc)_4 \xrightarrow{\text{Degradation}} null$$

$$(ub_{172}RIG-I_{uc}-unanc)_4 \xrightarrow{\text{Degradation}} null$$

$$(ub_{172}^{164}RIG-I_{uc}-unanc)_4 \xrightarrow{\text{Degradation}} null$$

1.2 Rate equations of RIG-I ubiquitination in random mechanism

$$V_1 = v_0 \cdot ([RIGI]-[RIGIuc])/(k_0+[RIGI]-[RIGIuc]) \cdot Riplet \cdot dsRNA$$

$$V_2 = d_1 \cdot [RIGI]$$

$$V_3 = v_3 \cdot [RIGIuc]/(k_3+[RIGIuc]) \cdot TRIM4$$

$$V_4 = v_1 \cdot [ub_{164}RIGIuc]/(k_1+[ub_{164}RIGIuc]) \cdot USPs$$

$$V_5 = v_4 \cdot [RIGIuc]/(k_4+[RIGIuc]) \cdot ([TRIM25]+\alpha \cdot [TRIM4])$$

$$V_6 = v_1 \cdot [ub_{172}RIGIuc]/(k_1+[ub_{172}RIGIuc]) \cdot USPs$$

$$V_7 = v_5 \cdot [RIGIuc]/(k_5+[RIGIuc]) \cdot [unanchoredUB]$$

$$V_8 = v_{1b} \cdot [RIGIuc\text{-}unanc]/(k_{1b}+[RIGIuc\text{-}unanc]) \cdot USPs$$

$$V_9 = v_{6a} \cdot [ub_{164}RIGIuc]/(k_{6a}+[ub_{164}RIGIuc]) \cdot ([TRIM25]+\alpha \cdot [TRIM4])$$

$$V_{10} = v_1 \cdot [ub_{172}^{164}RIGIuc]/(k_1+[ub_{172}^{164}RIGIuc]) \cdot USPs$$

$$V_{11} = v_{6b} \cdot [ub_{172}RIGIuc]/(k_{6b}+[ub_{172}RIGIuc]) \cdot [TRIM4]$$

$$V_{12} = v_1 \cdot [ub_{172}^{164}RIGIuc]/(k_1+[ub_{172}^{164}RIGIuc]) \cdot USPs$$

$$V_{13} = v_{7a} \cdot [ub_{164}RIGIuc]/(k_{7a}+[ub_{164}RIGIuc]) \cdot [unanchoredUB]$$

$$V_{14} = v_{1b} \cdot [ub_{164}RIGIuc\text{-}unanc]/(k_{1b}+[ub_{164}RIGIuc\text{-}unanc]) \cdot USPs$$

$$V_{15} = v_{8a} \cdot [ub_{172}RIGIuc]/(k_{8a}+[ub_{172}RIGIuc]) \cdot [unanchoredUB]$$

$$V_{16} = v_{1b} \cdot [ub_{172}RIGIuc\text{-}unanc]/(k_{1b}+[ub_{172}RIGIuc\text{-}unanc]) \cdot USPs$$

$$V_{17} = v_{7b} \cdot [RIGIuc\text{-}unanc]/(k_{7b}+[RIGIuc\text{-}unanc]) \cdot TRIM4$$

$$V_{18} = v_1 \cdot [ub_{164}RIGIuc\text{-}unanc]/(k_1+[ub_{164}RIGIuc\text{-}unanc]) \cdot USPs$$

$$V_{19} = v_{8b} \cdot [RIGIuc\text{-}unanc]/(k_{8b}+[RIGIuc\text{-}unanc]) \cdot ([TRIM25]+\alpha \cdot [TRIM4])$$

$$V_{20} = v_1 \cdot [ub_{172}RIGIuc\text{-}unanc]/(k_1+[ub_{172}RIGIuc\text{-}unanc]) \cdot USPs$$

$$V_{21} = v_{9a} \cdot [ub_{172}^{164}RIGIuc]/(k_{9a}+[ub_{172}^{164}RIGIuc]) \cdot [unanchoredUB]$$

$$V_{22} = v_{1b} \cdot \left[ub_{172}^{164}RIGIuc\text{-}unanc\right] / \left(k_{1b} + \left[ub_{172}^{164}RIGIuc\text{-}unanc\right]\right) \cdot USPs$$

$$V_{23} = v_{9b} \cdot \left[ub_{164}RIGIuc\text{-}unanc\right] / (k_{9b} + \left[ub_{164}RIGIuc\text{-}unanc\right]) \cdot \left([TRIM25] + \alpha \cdot [TRIM4]\right)$$

$$V_{24} = v_{1} \cdot \left[ub_{172}^{164}RIGIuc\text{-}unanc\right] / \left(k_{1} + \left[ub_{172}^{164}RIGIuc\text{-}unanc\right]\right) \cdot USPs$$

$$V_{25} = v_{9c} \cdot \left[ub_{172}RIGIuc\text{-}unanc\right] / \left(k_{9c} + \left[ub_{172}RIGIuc\text{-}unanc\right]\right) \cdot TRIM4$$

$$V_{26} = v_{1} \cdot \left[ub_{172}^{164}RIGIuc\text{-}unanc\right] / \left(k_{1} + \left[ub_{172}^{164}RIGIuc\text{-}unanc\right]\right) \cdot USPs$$

$$V_{27} = k_{m1} \cdot \left[ub_{164}RIGIuc\right]^4$$

$$V_{28} = k_{m2} \cdot \left[ub_{172}RIGIuc\right]^4$$

$$V_{29} = k_{m3} \cdot \left[RIGIuc\text{-}unanc\right]^4$$

$$V_{30} = k_{m4} \cdot \left[ub_{172}^{164}RIGIuc\right]^4$$

$$V_{31} = k_{m5} \cdot \left[ub_{164}RIGIuc\text{-}unanc\right]^4$$

$$V_{32} = k_{m6} \cdot \left[ub_{172}RIGIuc\text{-}unanc\right]^4$$

$$V_{33} = k_{m7} \cdot \left[ub_{172}^{164}RIGIuc\text{-}unanc\right]^4$$

$$V_{34} = d_2 \cdot \left[\left(:RIGIuc\bullet\right)_4\right]$$

Remark: $\left[\left(:RIGIuc\bullet\right)_4\right]$ in the above denotes the total concentration of various forms of tetramers of RIG-I, including $\left(ub_{164}RIGIuc\right)_4$, $\left(ub_{172}RIGIuc\right)_4$, $\left(RIGIuc\text{-}unanc\right)_4$, $\left(ub_{172}^{164}RIGIuc\right)_4$, $\left(ub_{164}RIGIuc\text{-}unanc\right)_4$, $\left(ub_{172}RIGIuc\text{-}unanc\right)_4$ and $\left(ub_{172}^{164}RIGIuc\text{-}unanc\right)_4$. The degradation rates of these tetramers were assumed to be the same (d_2).

1.3 ODEs model of RIG-I ubiquitination in random mechanism

$$\frac{d\left[RIGI\right]}{dt} = Vsys - V_1 - V_2$$

$$\frac{d\left[RIGIuc\right]}{dt}=V_1+V_4+V_6+V_8-V_3-V_5-V_7$$

$$\frac{d\left[ub_{164}RIGIuc\right]}{dt}=V_3-V_4-V_9+V_{10}-V_{13}+V_{14}-V_{27}$$

$$\frac{d\left[ub_{172}RIGIuc\right]}{dt}=V_5-V_6-V_{11}+V_{12}-V_{15}+V_{16}-V_{28}$$

$$\frac{d\left[RIGIuc\text{-}unanc\right]}{dt}=V_7-V_8-V_{17}+V_{18}-V_{19}+V_{20}-V_{29}$$

$$\frac{d\left[ub_{172}^{164}RIGIuc\right]}{dt}=V_9-V_{10}+V_{11}-V_{12}-V_{21}+V_{22}-V_{30}$$

$$\frac{d\left[ub_{164}RIGIuc\text{-}unanc\right]}{dt}=V_{13}-V_{14}+V_{15}-V_{18}-V_{23}+V_{24}-V_{31}$$

$$\frac{d\left[ub_{172}RIGIuc\text{-}unanc\right]}{dt}=V_{15}-V_{16}+V_{19}-V_{20}-V_{25}+V_{26}-V_{32}$$

$$\frac{d\left[ub_{172}^{164}RIGIuc\text{-}unanc\right]}{dt}=V_{21}-V_{22}+V_{23}-V_{24}+V_{25}-V_{33}$$

$$\frac{d\left[\left(:RIGIuc\bullet\right)_4\right]}{dt}=\sum_{i=27}^{33}V_i-V_{34}$$

1.4 ODEs of RIG-I downstream pathway in random mechanism

The regulations involved in the RIG-I downstream pathway MAVS-pIRF3-ISRE were modeled using Hill functions as follows:

$$\frac{d[MAVS]}{dt}=v_{11}\cdot\left[\left(:RIGIuc\mathrm{g}\right)_4\right]/\left(k11+\left[\left(:RIGIuc\mathrm{g}\right)_4\right]\right)-d_{11}\cdot[MAVS]$$

$$\frac{d\left[pIRF3\right]}{dt}=v_{12}\cdot\left(1-\left[pIRF3\right]\right)/\left(k_{12}+\left(1-\left[pIRF3\right]\right)\right)\cdot\left[MAVS\right]-d_{12}\cdot\left[pIRF3\right]$$

$$\frac{d\left[ISRE\right]}{dt}=v_{13}\cdot\left[pIRF3\right]/\left(k_{13}+\left[pIRF3\right]\right)-d_{13}\cdot\left[ISRE\right]$$

Text S2 Model formulation for sequential mechanism 1

2.1 Biochemical reactions of RIG-I ubiquitination in sequential mechanism 1

R_1 $\quad \mathrm{RIG-I} + \mathrm{dsRNA} \xrightarrow{\mathrm{Riplet}} \mathrm{RIG-I_{uc}}$

R_2 $\quad \mathrm{RIG-I} \xrightarrow{\mathrm{Degradation}} \mathrm{null}$

R_3 $\quad \mathrm{RIG-I_{uc}} \xrightarrow{\mathrm{TRIM4}} \mathrm{ub_{164}RIG-I_{uc}}$

R_4 $\quad \mathrm{ub_{164}RIG-I_{uc}} \xrightarrow{\mathrm{USPs}} \mathrm{RIG-I_{uc}}$

R_5 $\quad \mathrm{ub_{164}RIG-I_{uc}} \xrightarrow{\mathrm{TRIM4,TRIM25}} \mathrm{ub_{172}^{164}RIG-I_{uc}}$

R_6 $\quad \mathrm{ub_{172}^{164}RIG-I_{uc}} \xrightarrow{\mathrm{USPs}} \mathrm{ub_{164}RIG-I_{uc}}$

R_7 $\quad \mathrm{ub_{172}^{164}RIG-I_{uc}} \xrightarrow{\mathrm{unanchored\ UB}} \mathrm{ub_{172}^{164}RIG-I_{uc}-unanc}$

R_8 $\quad \mathrm{ub_{172}^{164}RIG-I_{uc}-unanc} \xrightarrow{\mathrm{USPs}} \mathrm{ub_{172}^{164}RIG-I_{uc}}$

R_9 $\quad 4 * \mathrm{ub_{164}RIG-I_{uc}} \rightarrow (\mathrm{ub_{164}RIG-I_{uc}})_4$

R_{10} $\quad 4 * \mathrm{ub_{172}^{164}RIG-I_{uc}} \rightarrow (\mathrm{ub_{172}^{164}RIG-I_{uc}})_4$

R_{11} $\quad 4 * \mathrm{ub_{172}^{164}RIG-I_{uc}-unanc} \rightarrow (\mathrm{ub_{172}^{164}RIG-I_{uc}-unanc})_4$

R_{12} $\quad (\mathrm{ub_{164}RIG-I_{uc}})_4 \xrightarrow{\mathrm{Degradation}} \mathrm{null}$

R_{13} $\quad (\mathrm{ub_{172}^{164}RIG-I_{uc}})_4 \xrightarrow{\mathrm{Degradation}} \mathrm{null}$

R_{14} $\quad (\mathrm{ub_{172}^{164}RIG-I_{uc}-unanc})_4 \xrightarrow{\mathrm{Degradation}} \mathrm{null}$

2.2 Rate equations of RIG-I ubiquitination in sequential mechanism 1

$$V_1 = v_0 \cdot ([RIGI]-[RIGIuc]) / (k_0 + [RIGI]-[RIGIuc]) \cdot Riplet \cdot dsRNA$$

$$V_2 = d_1 \cdot [RIGI]$$

$$V_3 = v_3 \cdot [RIGIuc] / (k_3 + [RIGIuc]) \cdot TRIM4$$

$$V_4 = v_1 \cdot [ub_{164}RIGIuc] / (k_1 + [ub_{164}RIGIuc]) \cdot USPs$$

$$V_5 = v_{5a} \cdot [ub_{164}RIGIuc] / (k_{5a} + [ub_{164}RIGIuc]) \cdot ([TRIM25] + \alpha \cdot [TRIM4])$$

$$V_6 = v_1 \cdot [ub_{172}^{164}RIGIuc] / (k_1 + [ub_{172}^{164}RIGIuc]) \cdot USPs$$

$$V_7 = v_{7a} \cdot [ub_{172}^{164}RIGIuc] / (k_{7a} + [ub_{172}^{164}RIGIuc]) \cdot [unanchoredUB]$$

$$V_8 = v_{1b} \cdot [ub_{172}^{164}RIGIuc\text{-}unanc] / (k_{1b} + [ub_{172}^{164}RIGIuc\text{-}unanc]) \cdot USPs$$

$$V_9 = k_{m1} \cdot [ub_{164}RIGIuc]^4$$

$$V_{10} = k_{m2} \cdot [ub_{172}^{164}RIGIuc]^4$$

$$V_{11} = k_{m3} \cdot [ub_{172}^{164}RIGIuc\text{-}unanc]^4$$

$$V_{12} = d_2 \cdot [(ub_{164}RIGIuc)_4]$$

$$V_{13} = d_3 \cdot [(ub_{172}^{164}RIGIuc)_4]$$

$$V_{14} = d_4 \cdot [(ub_{172}^{164}RIGIuc\text{-}unanc)_4]$$

2.3 ODEs model of RIG-I ubiquitination in sequential mechanism 1

$$\frac{d[RIGI]}{dt} = Vsys - V_1 - V_2$$

$$\frac{d[RIGIuc]}{dt} = V_1 - V_3 + V_4$$

$$\frac{d[ub_{164}RIGIuc]}{dt} = V_3 - V_4 - V_5 + V_6 - V_9$$

$$\frac{d[ub_{172}^{164}RIGIuc]}{dt} = V_5 - V_6 - V_7 + V_8 - V_{10}$$

$$\frac{d[ub_{172}^{164}RIGIuc\text{-}unanc]}{dt} = V_7 - V_8 - V_{11}$$

$$\frac{d\left[\left(ub_{164}RIGIuc\right)_4\right]}{dt}=V_9-V_{12}$$

$$\frac{d\left[\left(ub_{172}^{164}RIGIuc\right)_4\right]}{dt}=V_{10}-V_{13}$$

$$\frac{d\left[\left(ub_{172}^{164}RIGIuc\text{-}unanc\right)_4\right]}{dt}=V_{11}-V_{14}$$

2.4 ODEs model of RIG-I downstream pathway in sequential mechanism 1

The regulations involved in the RIG-I downstream pathway MAVS-pIRF3-ISRE were modeled using Hill functions as follows.

$$\frac{d[MAVS]}{dt}=k_6\cdot\left(\left[\left(ub_{164}RIGIuc\right)_4\right]+\left[\left(ub_{172}^{164}RIGIuc\right)_4\right]+\left[\left(ub_{172}^{164}RIGIuc-unanc\right)_4\right]\right)-d_6\cdot\left[MAVS\right]$$

$$\frac{d\left[pIRF3\right]}{dt}=v_6\cdot\left(1-\left[pIRF3\right]\right)/\left(k_{d1}+\left(1-\left[pIRF3\right]\right)\right)\cdot\left[MAVS\right]-d_7\cdot\left[pIRF3\right]$$

$$\frac{d\left[ISRE\right]}{dt}=v_7\cdot\left[pIRF3\right]/\left(k_{d2}+\left[pIRF3\right]\right)-d_8\cdot\left[ISRE\right]$$

Text S3 Model formulation for sequential mechanism 2

3.1 Biochemical reactions of RIG-I ubiquitination in sequential mechanism 2

Reactions R_1 - R_4 and R_9 - R_{14} were same with that in Text S2.1. The following are reactions different from that in sequential mechanism 1.

R_5 $\quad \mathrm{ub_{164}RIG-I_{uc}} \xrightarrow{\text{unanchored UB}} \mathrm{ub_{164}RIG-I_{uc}-unanc}$

R_6 $\quad \mathrm{ub_{164}RIG-I_{uc}-unanc} \xrightarrow{\text{USPs}} \mathrm{ub_{164}RIG-I_{uc}}$

R_7 $\quad \mathrm{ub_{164}RIG-I_{uc}-unanc} \xrightarrow{\text{TRIM4,TRIM25}} \mathrm{ub_{172}^{164}RIG-I_{uc}-unanc}$

R_8 $\quad \mathrm{ub_{172}^{164}RIG-I_{uc}-unanc} \xrightarrow{\text{USPs}} \mathrm{ub_{164}RIG-I_{uc}-unanc}$

3.2 Rate equations of RIG-I ubiquitination in sequential mechanism 2

Rate equations V_1-V_4 and V_9-V_{14} were same with that in Text S2.2. The following are rate equations different from that in sequential mechanism 1.

$$V_1 = v_0 \cdot \left(\left[RIGI\right]-\left[RIGIuc\right]\right) / \left(k_0 + \left[RIGI\right]-\left[RIGIuc\right]\right) \cdot Riplet \cdot dsRNA$$

$$V_2 = d_1 \cdot \left[RIGI\right]$$

$$V_3 = v_3 \cdot \left[RIGIuc\right] / \left(k_3 + \left[RIGIuc\right]\right) \cdot TRIM4$$

$$V_4 = v_1 \cdot \left[ub_{164}RIGIuc\right] / \left(k_1 + \left[ub_{164}RIGIuc\right]\right) \cdot USPs$$

$$V_5 = v_{5a} \cdot \left[ub_{164}RIGIuc\right] / \left(k_{5a} + \left[ub_{164}RIGIuc\right]\right) \cdot [unanchoredUB]$$

$$V_6 = v_{1b} \cdot \left[ub_{164}RIGIuc\text{-}unanc\right] / \left(k_{1b} + \left[ub_{164}RIGIuc\text{-}unanc\right]\right) \cdot USPs$$

$$V_7 = v_{7a} \cdot \left[ub_{164}RIGIuc\text{-}unanc\right] / \left(k_{7a} + \left[ub_{164}RIGIuc\text{-}unanc\right]\right) \cdot \left([TRIM25] + \alpha \cdot [TRIM4]\right)$$

$$V_8 = v_1 \cdot \left[ub_{172}^{164}RIGIuc\text{-}unanc\right] / \left(k_1 + \left[ub_{172}^{164}RIGIuc\text{-}unanc\right]\right) \cdot USPs$$

$$V_9 = k_{m1} \cdot \left[ub_{164}RIGIuc \right]^4$$

$$V_{10} = k_{m2} \cdot \left[ub_{164}RIGIuc\text{-}unanc \right]^4$$

$$V_{11} = k_{m3} \cdot \left[ub_{172}^{164}RIGIuc\text{-}unanc \right]^4$$

$$V_{12} = d_2 \cdot \left[\left(ub_{164}RIGIuc \right)_4 \right]$$

$$V_{13} = d_3 \cdot \left[ub_{164}RIGIuc\text{-}unanc \right]^4$$

$$V_{14} = d_4 \cdot \left[ub_{172}^{164}RIGIuc\text{-}unanc \right]^4$$

3.3 ODEs model of RIG-I ubiquitination in sequential mechanism 2

Same with that in Text S2.3 in the form $\frac{d[X_i]}{dt} = \sum V_+ - \sum V_-$.

3.4 ODEs model of RIG-I downstream pathway in sequential mechanism 2

The activation of MAVS was modeled as follows:

$$\frac{d[MAVS]}{dt} = k_6 \cdot \left(\left[\left(ub_{164}RIGIuc \right)_4 \right] + \left[\left(ub_{164}RIGIuc\text{-}unanc \right)_4 \right] + \left[\left(ub_{172}^{164}RIGIuc\text{-}unanc \right)_4 \right] \right) - d_6 \cdot \left[MAVS \right]$$

ODEs of pIRF3 and ISRE were same with that in Text S2.4.

Text S4 Model formulation for sequential mechanism 3

4.1 Biochemical reactions of RIG-I ubiquitination in sequential mechanism 3

Reactions R_1 - R_2 and R_9 - R_{14} were same with that in Text S2.1. The following are reactions different from that in sequential mechanism 1.

R_3 $\quad \mathrm{RIG-I_{uc}} \xrightarrow{\text{unanchored UB}} \mathrm{RIG-I_{uc}-unanc}$

R_4 $\quad \mathrm{RIG-I_{uc}-unanc} \xrightarrow{\text{USPs}} \mathrm{RIG-I_{uc}}$

R_5 $\quad \mathrm{RIG-I_{uc}-unanc} \xrightarrow{\text{TRIM4}} \mathrm{ub_{164}RIG-I_{uc}-unanc}$

R_6 $\quad \mathrm{ub_{164}RIG-I_{uc}-unanc} \xrightarrow{\text{USPs}} \mathrm{RIG-I_{uc}-unanc}$

R_7 $\quad \mathrm{ub_{164}RIG-I_{uc}-unanc} \xrightarrow{\text{TRIM4,TRIM25}} \mathrm{ub_{172}^{164}RIG-I_{uc}-unanc}$

R_8 $\quad \mathrm{ub_{172}^{164}RIG-I_{uc}-unanc} \xrightarrow{\text{USPs}} \mathrm{ub_{164}RIG-I_{uc}-unanc}$

4.2 Rate equations of RIG-I ubiquitination in sequential mechanism 3

Rate equations V_1-V_2 and V_9-V_{14} were same with that in Text S2.2. The following are rate equations different from that in sequential mechanism 1.

$$V_3 = v_{3a} \cdot [RIGIuc] / (k_{3a} + [RIGIuc]) \cdot [unanchoredUB]$$

$$V_4 = v_{1b} \cdot [RIGIuc\text{-}unanc] / (k_{1b} + [RIGIuc\text{-}unanc]) \cdot USPs$$

$$V_5 = v_5 \cdot [RIGIuc\text{-}unanc] / (k_5 + [RIGIuc\text{-}unanc]) \cdot TRIM4$$

$$V_6 = v_1 \cdot [ub_{164}RIGIuc\text{-}unanc] / (k_1 + [ub_{164}RIGIuc\text{-}unanc]) \cdot USPs$$

$$V_7 = v_{7a} \cdot [ub_{164}RIGIuc\text{-}unanc] / (k_{7a} + [ub_{164}RIGIuc\text{-}unanc]) \cdot ([TRIM25] + \alpha \cdot [TRIM4])$$

$$V_8 = v_1 \cdot [ub_{172}^{164}RIGIuc\text{-}unanc] / (k_1 + [ub_{172}^{164}RIGIuc\text{-}unanc]) \cdot USPs$$

4.3 ODEs model of RIG-I ubiquitination in sequential mechanism 3

Same with that in Text S2.3 in the form $\frac{d[X_i]}{dt} = \sum V_+ - \sum V_-$.

4.4 ODEs model of RIG-I downstream pathway in sequential mechanism 3

The activation of MAVS was modeled as follows:

$$\frac{d[MAVS]}{dt} = k_6 \cdot \left(\left[\left(RIGIuc\text{-}unanc \right)_4 \right] + \left[\left(ub_{164} RIGIuc\text{-}unanc \right)_4 \right] + \left[\left(ub_{172}^{164} RIGIuc\text{-}unanc \right)_4 \right] \right) - d_6 \cdot \left[MAVS \right]$$

ODEs of pIRF3 and ISRE were same with that in Text S2.4.

Text S5 Model formulation for hierarchical mechanism 1

5.1 Biochemical reactions of RIG-I ubiquitination in hierarchical mechanism 1

R_1 $\quad \mathrm{RIG-I} + \mathrm{dsRNA} \xrightarrow{\mathrm{Riplet}} \mathrm{RIG-I_{uc}}$

R_2 $\quad \mathrm{RIG-I} \xrightarrow{\mathrm{Degradation}} \mathrm{null}$

R_3 $\quad \mathrm{RIG-I_{uc}} \xrightarrow{\mathrm{TRIM4}} \mathrm{ub_{164}RIG-I_{uc}}$

R_4 $\quad \mathrm{ub_{164}RIG-I_{uc}} \xrightarrow{\mathrm{USPs}} \mathrm{RIG-I_{uc}}$

R_5 $\quad \mathrm{RIG-I_{uc}} \xrightarrow{\mathrm{TRIM4,TRIM25}} \mathrm{ub_{172}RIG-I_{uc}}$

R_6 $\quad \mathrm{ub_{172}RIG-I_{uc}} \xrightarrow{\mathrm{USPs}} \mathrm{RIG-I_{uc}}$

R_7 $\quad \mathrm{ub_{164}RIG-I_{uc}} \xrightarrow{\mathrm{TRIM4,TRIM25}} \mathrm{ub_{172}^{164}RIG-I_{uc}}$

R_8 $\quad \mathrm{ub_{172}RIG-I_{uc}} \xrightarrow{\mathrm{TRIM25}} \mathrm{ub_{172}^{164}RIG-I_{uc}}$

R_9 $\quad \mathrm{ub_{172}^{164}RIG-I_{uc}} \xrightarrow{\mathrm{USPs}} \mathrm{ub_{164}RIG-I_{uc}}$ or $\mathrm{ub_{172}RIG-I_{uc}}$

R_{10} $\quad \mathrm{ub_{172}^{164}RIG-I_{uc}} \xrightarrow{\text{unanchored UB}} \mathrm{ub_{172}^{164}RIG-I_{uc}} - unanc$

R_{11} $\quad \mathrm{ub_{172}^{164}RIG-I_{uc}} - uanc \xrightarrow{\mathrm{USPs}} \mathrm{ub_{172}^{164}RIG-I_{uc}}$

R_{12} $\quad 4 * \mathrm{ub_{164}RIG-I_{uc}} \rightarrow (\mathrm{ub_{164}RIG-I_{uc}})_4$

R_{13} $\quad 4 * \mathrm{ub_{172}RIG-I_{uc}} \rightarrow (\mathrm{ub_{172}RIG-I_{uc}})_4$

R_{14} $\quad 4 * \mathrm{ub_{172}^{164}RIG-I_{uc}} \rightarrow (\mathrm{ub_{172}^{164}RIG-I_{uc}})_4$

R_{15} $\quad 4 * \mathrm{ub_{172}^{164}RIG-I_{uc}} - unanc \rightarrow (\mathrm{ub_{172}^{164}RIG-I_{uc}} - unanc)_4$

R_{16} $\quad (\mathrm{ub_{164}RIG-I_{uc}})_4 \xrightarrow{\mathrm{Degradation}} \mathrm{null}$

R_{17} $\quad (\mathrm{ub_{172}RIG-I_{uc}})_4 \xrightarrow{\mathrm{Degradation}} \mathrm{null}$

R_{18} $\quad (\mathrm{ub_{172}^{164}RIG-I_{uc}})_4 \xrightarrow{\mathrm{Degradation}} \mathrm{null}$

R_{19} $\quad (\mathrm{ub_{172}^{164}RIG-I_{uc}} - unanc)_4 \xrightarrow{\mathrm{Degradation}} null$

5.2 Rate equations of RIG-I ubiquitination in hierarchical mechanism 1

$$V_1 = v_1 \cdot ([RIGI]-[RIGIuc]) / (k_{m1} + [RIGI]-[RIGIuc]) \cdot Riplet \cdot dsRNA$$

$$V_2 = d_1 \cdot [RIGI]$$

$$V_3 = v_2 \cdot [RIGIuc] / (k_{m2} + [RIGIuc]) \cdot TRIM4$$

$$V_4 = v_5 \cdot [ub_{164}RIGIuc] / (k_{m5} + [ub_{164}RIGIuc]) \cdot USPs$$

$$V_5 = v_3 \cdot [RIGIuc] / (k_{m3} + [RIGIuc]) \cdot (TRIM25 + \alpha \cdot TRIM4)$$

$$V_6 = v_5 \cdot [ub_{172}RIGIuc] / (k_{m5} + [ub_{172}RIGIuc]) \cdot USPs$$

$$V_7 = v_{4a} \cdot [ub_{164}RIGIuc] / (k_{m4a} + [ub_{164}RIGIuc]) \cdot (TRIM25 + \alpha \cdot TRIM4)$$

$$V_8 = v_{4b} \cdot [ub_{172}RIGIuc] / (k_{m4b} + [ub_{172}RIGIuc]) \cdot TRIM4$$

$$V_9 = v_5 \cdot [ub_{172}^{164}RIGIuc] / (k_{m5} + [ub_{172}^{164}RIGIuc]) \cdot USPs$$

$$V_{10} = K_5 \cdot [ub_{172}^{164}RIGIuc] \cdot [unanchoredUB]$$

$$V_{11} = v_5 \cdot [ub_{172}^{164}RIGIuc\text{-}unanc] / (k_{m5} + [ub_{172}^{164}RIGIuc\text{-}unanc]) \cdot USPs$$

$$V_{12} = k_1 \cdot [ub_{164}RIGIuc]^4$$

$$V_{13} = k_2 \cdot [ub_{172}RIGIuc]^4$$

$$V_{14} = k_3 \cdot [ub_{172}^{164}RIGIuc]^4$$

$$V_{15} = k_4 \cdot [ub_{172}^{164}RIGIuc\text{-}unanc]^4$$

$$V_{16} = d_2 \cdot [(ub_{164}RIGIuc)_4]$$

$$V_{17} = d_3 \cdot [(ub_{172}RIGIuc)_4]$$

$$V_{18} = d_4 \cdot [(ub_{172}^{164}RIGIuc)_4]$$

$$V_{19} = d_5 \cdot [(ub_{172}^{164}RIGIuc\text{-}unanc)_4]$$

5.3 ODEs model of RIG-I ubiquitination in hierarchical mechanism 1

$$\frac{d\left[RIGI\right]}{dt} = Vsys - V_1 - V_2$$

$$\frac{d\left[RIGIuc\right]}{dt} = V_1 + V_4 + V_6 - V_3 - V_5$$

$$\frac{d\left[ub_{164}RIGIuc\right]}{dt} = V_3 - V_4 - V_7 + V_9 - V_{12}$$

$$\frac{d\left[ub_{172}RIGIuc\right]}{dt} = V_5 - V_6 - V_8 + V_9 - V_{13}$$

$$\frac{d\left[ub_{172}^{164}RIGIuc\right]}{dt} = V_7 + V_8 - V_9 - V_{10} + V_{11} - V_{14}$$

$$\frac{d\left[ub_{172}^{164}RIGIuc\text{-}unanc\right]}{dt} = V_{10} - V_{11} - V_{15}$$

$$\frac{d\left[\left(ub_{164}RIGIuc\right)_4\right]}{dt} = V_{12} - V_{16}$$

$$\frac{d\left[\left(ub_{172}RIGIuc\right)_4\right]}{dt} = V_{13} - V_{17}$$

$$\frac{d\left[\left(ub_{172}^{164}RIGIuc\right)_4\right]}{dt} = V_{14} - V_{18}$$

$$\frac{d\left[\left(ub_{172}^{164}RIGIuc\text{-}unanc\right)_4\right]}{dt} = V_{15} - V_{19}$$

5.4 ODEs model of RIG-I downstream pathway in hierarchical mechanism 1

The regulations involved in the RIG-I downstream pathway MAVS-pIRF3-ISRE were modeled using Hill functions as follows.

$$\frac{d[MAVS]}{dt} = k_6 \cdot \Big(\left[\left(ub_{164}RIGIuc\right)_4\right] + \left[\left(ub_{172}RIGIuc\right)_4\right] + \left[\left(ub_{172}^{164}RIGIuc\right)_4\right]$$

$$+ \left[\left(ub_{172}^{164}RIGIuc - unanc\right)_4\right] \Big) - d_6 \cdot [MAVS]$$

$$\frac{d[pIRF3]}{dt} = v_6 \cdot (1 - [pIRF3]) / (k_{d1} + (1 - [pIRF3])) \cdot [MAVS] - d_7 \cdot [pIRF3]$$

$$\frac{d[ISRE]}{dt} = v_7 \cdot [pIRF3] / (k_{d2} + [pIRF3]) - d_8 \cdot [ISRE]$$

Text S6 Model formulation for hierarchical mechanism 2

6.1 Biochemical reactions of RIG-I ubiquitination in hierarchical mechanism 2

Reactions R_1-R_2 and R_{12} -R_{19} were same with that in Text S5.1. The following are reactions different from that in sequential mechanism 1.

R_3 $\mathrm{RIG-I_{uc}} \xrightarrow{\text{unanchored UB}} \mathrm{RIG-I_{uc}-unanc}$

R_4 $\mathrm{RIG-I_{uc}-unanc} \xrightarrow{\text{USPs}} \mathrm{RIG-I_{uc}}$

R_5 $\mathrm{RIG-I_{uc}-unanc} \xrightarrow{\text{TRIM 4}} \mathrm{ub_{164}RIG-I_{uc}-unanc}$

R_6 $\mathrm{ub_{164}RIG-I_{uc}-unanc} \xrightarrow{\text{USPs}} \mathrm{RIG-I_{uc}-unanc}$

R_7 $\mathrm{RIG-I_{uc}-unanc} \xrightarrow{\text{TRIM 4,TRIM 25}} \mathrm{ub_{172}RIG-I_{uc}-unanc}$

R_8 $\mathrm{ub_{172}RIG-I_{uc}-unanc} \xrightarrow{\text{USPs}} \mathrm{RIG-I_{uc}-unanc}$

R_9 $\mathrm{ub_{164}RIG-I_{uc}-unanc} \xrightarrow{\text{TRIM 4,TRIM 25}} \mathrm{ub_{172}^{164}RIG-I_{uc}-unanc}$

R_{10} $\mathrm{ub_{172}RIG-I_{uc}-unanc} \xrightarrow{\text{TRIM 25}} \mathrm{ub_{172}^{164}RIG-I_{uc}-unanc}$

R_{11} $\mathrm{ub_{172}^{164}RIG-I_{uc}-unanc} \xrightarrow{\text{USPs}} \mathrm{ub_{164}RIG-I_{uc}-unanc}$ or $\mathrm{ub_{172}RIG-I_{uc}-unanc}$

6.2 Rate equations of RIG-I ubiquitination in hierarchical mechanism 2

Rate equations V_1-V_2 and V_{12}-V_{19} were same with that in Text S5.2. The following are rate equations different from that in sequential mechanism 1.

$$V_3 = k_1 \cdot [RIGIuc] \cdot [unanchoredUB]$$

$$V_4 = v_5 \cdot [RIGIuc\text{-}unanc] / (k_{m5} + [RIGIuc\text{-}unanc]) \cdot USPs$$

$$V_5 = v_2 \cdot [RIGIuc\text{-}unanc] / (k_{m2} + [RIGIuc\text{-}unanc]) \cdot TRIM4$$

$$V_6 = v_5 \cdot \left[ub_{164}RIGIuc\text{-}unanc\right] / (k_{m5} + \left[ub_{164}RIGIuc\text{-}unanc\right]) \cdot USPs$$

$$V_7 = v_3 \cdot \left[RIGIuc\text{-}unanc\right] / (k_{m3} + \left[RIGIuc\text{-}unanc\right]) \cdot \left([TRIM25] + \alpha \cdot [TRIM4]\right)$$

$$V_8 = v_5 \cdot \left[ub_{172}RIGIuc\text{-}unanc\right] / (k_{m5} + \left[ub_{172}RIGIuc\text{-}unanc\right]) \cdot USPs$$

$$V_9 = v_{4a} \cdot \left[ub_{164}RIGIuc\text{-}unanc\right] / (k_{m4a} + \left[ub_{164}RIGIuc\text{-}unanc\right]) \cdot \left([TRIM25] + \alpha \cdot [TRIM4]\right)$$

$$V_{10} = v_{4b} \cdot \left[ub_{172}RIGIuc\text{-}unanc\right] / (k_{m4b} + \left[ub_{172}RIGIuc\text{-}unanc\right]) \cdot TRIM4$$

$$V_{11} = v_5 \cdot \left[ub_{172}^{164}RIGIuc\text{-}unanc\right] / (k_{m5} + \left[ub_{172}^{164}RIGIuc\text{-}unanc\right]) \cdot USPs$$

6.3 ODEs model of RIG-I ubiquitination in hierarchical mechanism 2

$$\frac{d\left[RIGI\right]}{dt} = Vsys - V_1 - V_2$$

$$\frac{d\left[RIGIuc\right]}{dt} = V_1 + V_4 - V_3$$

$$\frac{d\left[RIGIuc\text{-}unanc\right]}{dt} = V_3 + V_4 - V_5 - V_7 + V_6 + V_8$$

$$\frac{d\left[ub_{164}RIGIuc\text{-}unanc\right]}{dt} = V_5 - V_6 - V_9 + V_{11}$$

$$\frac{d\left[ub_{172}RIGIuc\text{-}unanc\right]}{dt} = V_7 - V_8 - V_{10} + V_{11}$$

$$\frac{d\left[ub_{172}^{164}RIGIuc\text{-}unanc\right]}{dt} = V_9 + V_{10} - V_{11}$$

ODEs of various forms of tetramers of RIG-I, including $\left(RIGIuc\text{-}unanc\right)_4$, $\left(ub_{164}RIGIuc\text{-}unanc\right)_4$, $\left(ub_{172}RIGIuc\text{-}unanc\right)_4$ and $\left(ub_{172}^{164}RIGIuc\text{-}unanc\right)_4$ were same with that in Text S5.3.

6.4 ODEs model of RIG-I downstream pathway in hierarchical mechanism 2

The activation of MAVS was modeled as follows:

$$\frac{d[MAVS]}{dt} = k_6 \cdot \Big(\big[(RIGIuc\text{-}unanc)_4 \big] + \big[(ub_{164}RIGIuc\text{-}unanc)_4 \big] + \big[(ub_{172}RIGIuc\text{-}unanc)_4 \big]$$

$$+ \big[(ub_{172}^{164}RIGIuc\text{-}unanc)_4 \big] \Big) - d_6 \cdot [MAVS]$$

ODEs of pIRF3 and ISRE were same with that in Text S5.4.

Text S7 Model formulation for hierarchical mechanism 3

7.1 Biochemical reactions of RIG-I ubiquitination in hierarchical mechanism 3

Same with that in hierarchical mechanism 1 (Text S5).

7.2 Rate equations of RIG-I ubiquitination in hierarchical mechanism 3

Same with that in hierarchical mechanism 1 (Text S5).

7.3 ODEs model of RIG-I ubiquitination in hierarchical mechanism 3

Same with that in hierarchical mechanism 1 (Text S5).

7.4 ODEs model of RIG-I downstream pathway in hierarchical mechanism 3

The activation of MAVS was modeled as follows:

$$\frac{d[MAVS]}{dt} = k_6 \cdot \left(\left[\left(ub_{172}^{164} RIGIuc \right)_4 \right] + \left[\left(ub_{172}^{164} RIGIuc\text{-}unanc \right)_4 \right] \right) - d_6 \cdot [MAVS]$$

ODEs of pIRF3 and ISRE were same with that in Text S5.4.

Text S8 Optimization algorithm

As described in the main text, our experimental data included time course data and dose response data under 4 different conditions: WT, K164R, K172R and k164&172R, that were represented by 4 binary variables incorporated in the mathematical model. Such types of data are much informative necessarily to reflect the kinetics of the signaling system. In order to simultaneously fit unknown parameters in the model to the conditions-dependent time course data and dose response data simultaneously, we modified a genetic algorithm associated with a conditions-dependent 4th Runge-Kutta method to solve the system of ODEs. The detailed optimization algorithm used to estimate parameters was described as follows.

1. Initialization. Set generation index $t=1$. Randomly generate the initial population $P(0)=\{P_1(0),\ldots, P_N(0)\}$ within the parameter space Θ, N is the population size.

2. Repeat the following procedures (2.1-2.5) until $t> T$ or $f(t)$<error to track best individual.

2.1 Numerically solve conditions-dependent ODEs with parameter values $P_i(t)$ as below,

$$Y^{sim}\left(cond_i, dose_j, t_k; P_i(t)\right) = ODEsolver(Y_0, P_i(t), Cond_i, dose_j, t_k \in tspan),$$

where Y_0 is a set of initial values of variables in the ODEs. Binary variables $Cond_i$ (i=1,2,3,4) indicate 4 different conditions. $dose_j$ is the dose of the stimulus. ODEs was numerically solved using the 4th Runge-Kutta method. The simulated response, Y^{sim}, was evaluated at a series of time points t_k in the range of $tspan$.

2.2 Return the calculated Y^{sim} to the objective function to evaluate the fitness $\{f_1(t),\ldots, f_N(t)\}$ of all individuals in P(t), and record the smallest fitness $f(t)$.

2.3 Generate a next generation population $P(t+1) =\{P_1(t+1),\ldots, P_N(t+1)\}$ from those selected through a combination of genetic operators: crossover and mutation.

2.4 Repeat above steps (2.2, 2.3) to calculate the fitness for N new individuals, and record the smallest fitness $f(t+1)$ of the population $P(t+1)$.

2.5 Update generation index $t := t+1$.

3. Record the individuals with the smallest fitness as the optimized parameters.

Text S9 Parameter sensitivity analysis

Parameters sensitivity analysis was performed for the estimated parameters (see Materials and Methods) to investigate which parameters in the model are more sensitive or critical. Time-averaged sensitivity coefficient [33, 34] of a variable y_i at time *t* with respect to a parameter P_j was calculated according to

$$S_{ij} = \frac{1}{T}\int_0^T abs\left(\frac{\partial y_i(t)}{\partial P_j} \middle/ \frac{y_i(t)}{P_j} \right) dt,$$

A small perturbation (e.g. ΔP_j=1%) was increased for each parameter from its estimated value.

Table S1 Quantification of robustness indexes of 7 models

The following table lists the robustness indexes of 7 models to various perturbations (K164R; K172R; no linkage of unanchored ubiquitin chains, K164, 172R; K164 only; K172 only). For each model, the average of robustness with respect to 6 perturbations was also calculated.

	Perturbations						Average
	K164R	K172R	No unanchored Ub	K164/172R	K164 only	K172 only	
Random	0.7758	0.7421	0.88	0.6377	0.1656	0.1491	0.5584
Sequential 1	0	0.4893	0	0	0.4893	0	0.1631
Sequential 2	0	0.9996	0.3897	0	0	0	0.2316
Sequential 3	0.2319	0.0101	1	0.2319	0.0101	0.2319	0.286
Hierarchical 1	0.6071	0.6315	0.6433	0	0.6315	0.6071	0.5201
Hierarchical 2	0.9827	0.763	0	0	0	0	0.291
Hierarchical 3	0	0	0.7897	0	0	0	0.1316

Table S2 Estimated parameter values

The following table lists the estimated values of parameters involved in the ODE model of hierarchical mechanism 1.

Para No.	Symbol	Value	Description
1	v_{sys}	0.564706	basal expression rate of RIG-I
2	v_1	0.584314	maximal activation rate of RIG-I by Riplet
3	v_2	0.341176	maximal ubiquitination rate of RIG-I at K164 by TRIM4
4	v_3	0.227451	maximal ubiquitination rate of RIG-Iuc at K172 by TRIM25
5	v_{4a}	0.058824	maximal ubiquitination rate of ub164RIG-I at K172 by TRIM25
6	v_{4b}	0.145098	maximal ubiquitination rate of ub172RIG-I at K164 by TRIM4
7	k_{m1}	0.545098	Michaelis constant of ubiquitination of RIG-I by Riplet
8	k_{m2}	0.768627	Michaelis constant of ubiquitination of RIG-I at K164 by TRIM4
9	k_{m3}	0.019608	Michaelis constant of ubiquitination of RIG-I at K172 by TRIM25
10	k_{m4a}	0.682353	Michaelis constant of ubiquitination of ub164RIG-I at K172 by TRIM25
11	k_{m4b}	0.231373	Michaelis constant of ubiquitination of ub172RIG-I at K164 by TRIM4
12	v_5	0.043137	maximal deubiquitination rate of RIG-I by USPs
13	k_{m5}	0.658824	Michaelis constant of deubiquitination of RIG-I by USPs
14	K_5	0.858824	ubiquitination rate of ub164_172RIG-Iuc by unanchored UB chain
15	k_1	0.360784	oligomerization rate of ub164RIG-I
16	k_2	0.835294	oligomerization rate of ub172RIG-I
17	k_3	0.431373	oligomerization rate of ub164_172RIG-I
18	k_4	0.039216	oligomerization rate of ub164_172RIG-I_unanchoreUB
19	k_5	0.772549	activation rate of MAVS by RIG-I oligomerization
20	v_6	0.909804	maximal activation rate of pIRF3 by MAVS
21	v_7	0.486275	maximal activation rate of ISRE by pIRF3
22	k_{d1}	0.772549	Michaelis constant of activation of pIRF3 by MAVS
23	k_{d2}	0.282353	Michaelis constant of activation of ISRE by pIRF3
24	d_6	0.839216	degradation rate of MAVS
25	d_8	0.498039	degradation rate of ISRE
26	α	0.500000	relative coefficient of TRIM4 action on K164 ubiquitination with respect to that by TRIM25

Table S3 Experimentally measured degradation rates

The following table lists the values of some degradation rates measured from the experimental data.

Para No.	Symbol	Value	Description
1	d_1	0.103534	degradation rate of RIG-I
2	d_2	0.729859	degradation rate of RIG-Iuc
3	d_3	0.729859	degradation rate of ub164RIG-Iuc
4	d_4	0.729859	degradation rate of ub172RIG-Iuc
5	d_5	0.729859	degradation rate of ub164-172RIG-Iuc
6	d_7	0.286780	degradation rate of pIRF3
